# Tree-Based QSAR Model for Drug Repurposing in the Discovery of New Antibacterial Compounds against *Escherichia coli*

**DOI:** 10.3390/ph13120431

**Published:** 2020-11-28

**Authors:** Beatriz Suay-Garcia, Antonio Falcó, J. Ignacio Bueso-Bordils, Gerardo M. Anton-Fos, M. Teresa Pérez-Gracia, Pedro A. Alemán-López

**Affiliations:** 1 Departamento de Matemáticas, Física y Ciencias Tecnológicas, Universidad Cardenal Herrera-CEU, CEU Universities, Alfara del Patriarca, 46115 Valencia, Spain; 2 Departamento de Farmacia Universidad Cardenal Herrera-CEU, CEU Universities, Alfara del Patriarca, 46115 Valencia, Spain; jose.bueso@uchceu.es (J.I.B.-B.); ganton@uchceu.es (G.M.A.-F.); teresa@uchceu.es (M.T.P.-G.); paleman@uchceu.es (P.A.A.-L.)

**Keywords:** drug repurposing, QSAR, antibiotics, drug development, *Escherichia coli*

## Abstract

Drug repurposing appears as an increasing popular tool in the search of new treatment options against bacteria. In this paper, a tree-based classification method using Linear Discriminant Analysis (LDA) and discrete indexes was used to create a QSAR (Quantitative Structure-Activity Relationship) model to predict antibacterial activity against *Escherichia coli*. The model consists on a hierarchical decision tree in which a discrete index is used to divide compounds into groups according to their values for said index in order to construct probability spaces. The second step consists in the calculation of a discriminant function which determines the prediction of the model. The model was used to screen the DrugBank database, identifying 134 drugs as possible antibacterial candidates. Out of these 134 drugs, 8 were antibacterial drugs, 67 were drugs approved for different pathologies and 55 were drugs in experimental stages. This methodology has proven to be a viable alternative to the traditional methods used to obtain prediction models based on LDA and its application provides interesting new drug candidates to be studied as repurposed antibacterial treatments. Furthermore, the topological indexes *Nclass* and *Numhba* have proven to have the ability to group active compounds effectively, which suggests a close relationship between them and the antibacterial activity of compounds against *E. coli*.

## 1. Introduction

Until the 1950s, drug development was a costly and time-consuming process based on experimental trial and error assays. The aim was to find a lead molecule by testing a group of compounds, both synthetic and natural, for a given pharmacological activity. Once the lead compound was identified, structural modifications were carried out to improve its properties [1]. In order to reduce the cost and time involved in drug discovery, QSAR (Quantitative Structure-Activity Relationship) methods were developed [2]. These methods marked a turning point in drug research, since they are able to predict the pharmacological activity of a molecule without having to synthesize or extract it. Thus, Computational Chemistry and Virtual Screening have become essential strategies for drug development [3].

In this context, machine learning (ML) has emerged as a key tool in pharmaceutical research, including bioactivity prediction, *de novo* molecular design, synthesis prediction and biological image analysis [4]. In fact, machine learning can aid several steps of the drug discovery process: prediction of target structure, construction of models to predict biological activity, discovery and optimization of lead compounds and prediction of pharmacokinetic and toxicological profiles [5,6]. Among the machine learning strategies available, tree-based methods or, CART (Classification and Regression Trees), appear as a powerful and popular option for the development of QSAR methods, seeing as they can be used for both classification and regression depending on the nature of the variable [7]. Variations of CART, including Random Forests (RF), have been used for the development of HIV [8], cancer [9] and Alzheimer’s disease [10] treatments. These models act by stratifying or segmenting the predictor space into a number of simpler regions. As a result, each observation belongs to the most commonly occurring class of training observations in the region to which it belongs [11]. These methods, combined with molecular topology, constitute a time- and cost-effective option to tackle drug discovery. In fact, molecular topology is especially interesting because the models built using molecular topology indices can be applied to databases with structural diversity, as the selection of active compounds is carried out taking only into account the mathematical-topological similarity [12]. As a consequence, the theoretically active compounds selected by these models will possess similar pharmacokinetic and pharmacodynamic properties, regardless of its structural family.

There are newer LBVS (ligand-based virtual screening) approaches, such as multitasking QSAR (mt-QSAR) [13,14] and multi-objective optimization QSAR (MOOP) [15,16] that aim to integrate multiple diverse chemical and biological data. In this sense, these models are capable of making predictions ranging from in vitro and in vivo activities to ADMET properties in order to obtain the drug candidate with not only the best activity but also the best pharmacokinetic and pharmacodynamic properties. These QSAR models are often built using ML techniques such as the one used in this paper [15]. However, mt-QSAR and MOOP QSAR were not considered for this paper as the goal was to obtain a highly precise predictive model capable of identifying candidates within a database of drugs that have already been characterized for these properties, to be repurposed as antibacterial agents against *E. coli.*

The main limiting factor in drug development and, especially in the development of antibacterial compounds, is its economic cost. This is an important deterrent for the pharmaceutical industry, seeing as investing said time and resources in an antibiotic which, besides being a short duration acute treatment, may have resistances appear withing a few years after implementing the treatment, does not appear as a profitable investment [17]. This is particularly important in the case of antibacterial drugs, for which the pharmaceutical industry has lost its interest [18]. Firstly, the increasing and apparently unstoppable appearance of antibiotic resistance results in a constant demand of new antibacterial agents. Furthermore, the identification of active compounds with structural diversity outside of the known antibacterial families could imply the discovery of new mechanisms of action, which could contribute to slow down the issue of antibiotic resistance [19]. 

Along these lines, drug repurposing offers an alternative method for the fast and cost-effective identification of new therapeutic agents [20,21]. The first examples of this approach date back to the 80s, when sildenafil, which was originally developed for chest angina, was finally marketed for erectile disfunction [22]. Another example is azidothymidine which, having failed as an antineoplastic drug, it was repurposed as a successful HIV treatment [23]. By using drug repurposing to discover new compounds with antibacterial activity, this paper aims to find a time- and cost-effective way to tackle the ever-growing issue of antibiotic resistance. Prior success stories such as those of sildenafil or azidothymidine encourage this work, since they prove the viability of this approach. 

The main aim of this paper is to develop a tree-based QSAR model using Linear Discriminant Analysis (LDA) and discrete indexes for the screening of DrugBank [24] in order to identify drug candidates to be repurposed as antibacterial compounds against *Escherichia coli* infection. 

## 2. Results

### 2.1. Tree-Based QSAR Model

#### 2.1.1. Compound Selection and Index Calculation

A total of 82 molecules (Supplementary Material, Tables S1 and S2) belonging to the quinolone family of antibacterial compounds were selected and divided into two groups, 43 compounds with proven antibacterial activity and 39 compounds described as inactive against *E. coli*. In order to consider a compound as active, its minimum inhibitory concentration (MIC) had to be ≤ 1 mg/L. On the other hand, a compound was considered inactive when it had a MIC > 16 mg/L. The compounds with MIC values in the 1–16 mg/L range were not included in the study because the property studied is biological and the relationship with the chemical structure is a matter of probability. Therefore, we wanted to make a notable difference between groups in order to be able to identify relevant structural references among active and inactive compounds. Furthermore, in the case of molecules with chiral centers, we only included those where all the stereoisomers were described as active, seeing as all stereoisomers are represented by the same chemical graph.

#### 2.1.2. Discrete Index Analysis

Firstly, we analyzed the probability distribution of 15 different discrete indexes calculated for our group of molecules in order to determine if any of them had good discriminant power (Supplementary Material, Figure S1)

**Figure 1:**
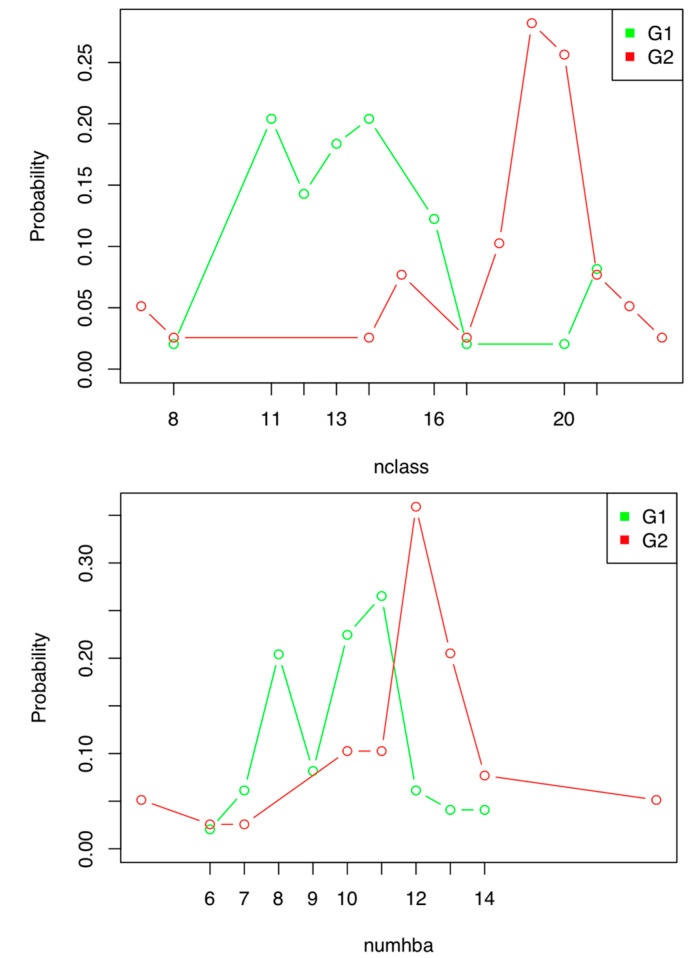
Distribution diagram of the active (G1) and inactive (G2) compounds used to construct the antibiotic activity prediction model against *E. coli* according to their *Nclass* (**up**) and *Numhba* (**down**) values.

When analyzing the distribution diagrams, two discrete indexes showed good discriminant power: *Nclass* and *Numhba* (Figure 1). However, *Nclass* was discarded because, when the compounds used to build the model were grouped according to their values for this index, it resulted in two groups for which DFs could not be calculated. This is due to the fact that the statistical program was chosen as the first step of the hierarchical decision tree, grouping the active compounds in the value range of 7–11 (Figure 1).

#### 2.1.3. Probability Space Construction

After selecting the discrete index, we grouped the compounds used for the construction of the model according to their *Numhba* values, resulting in 2 groups:

- *Numhba* = 7–11
- *Numhba*≠ 7–11

When analyzing the resulting groups, we observed that there were no active compounds among the molecules with values of *Numhba* ≠ 7–11, therefore, we decided that compounds with values of *Numhba* outside of the 7–11 range would be directly classified as inactive. Thus, only one discriminant function was calculated for the group of compounds with *Numhba* values within the 7–11 range.

#### 2.1.4. Linear Discriminant Analysis

Once the probability spaces had been established, a DF, presented below along with its corresponding statistical parameters, was calculated for the maximum probability space using continuous indexes and LDA:

DF = −129.49658 − 16.29048*Phia* + 16.10739*SH_tCH_* + 3.57226*S_=N−_* − 5.11438*S_>N−_* − 25.95896*^4^C_C_* (1)

N = 48; λ = 0.1065407; F = 50.317 

**Figure 2:**
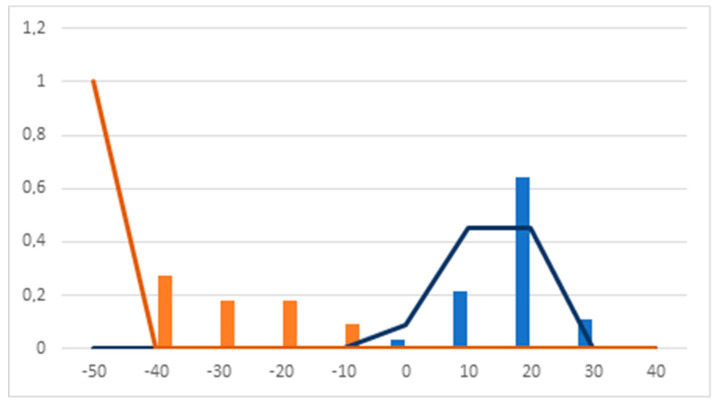
Pharmacological distribution diagram of the discriminant function (DF). (Orange bars: inactive training. Blue bars: active training. Orange lines: inactive test. Blue lines: active test).

Analyzing the Pharmacological Distribution Diagram (PDD) for the calculated DF (Figure 2), it can be observed that active compounds lie in the 0–40 interval. On the other hand, compounds with DF values of < 0 and ≥ −50 are classified as inactive. Compounds with DF values < 40 and > −50 remain unclassifiable. As a consequence, the PDD defines the applicability domain of the model, which is limited to compounds with DF values between −50 and 40.

As it can be observed in Table 1, the model had a 100% accuracy classification rate using the training and test sets. Furthermore, due to the natural imbalance of the training and test sets, the Matthew’s Correlation Coefficient (MCC) was calculated, obtaining and MCC value of 1.

Tables providing information regarding the value of each of the indexes present in the DF, the value of the DF for each compound, the classification probability for each compound and their final classification as active or inactive are provided as Supplementary Material (Tables S3–S6).

#### 2.1.5. Hierarchical Tree Construction

**Figure 3:**
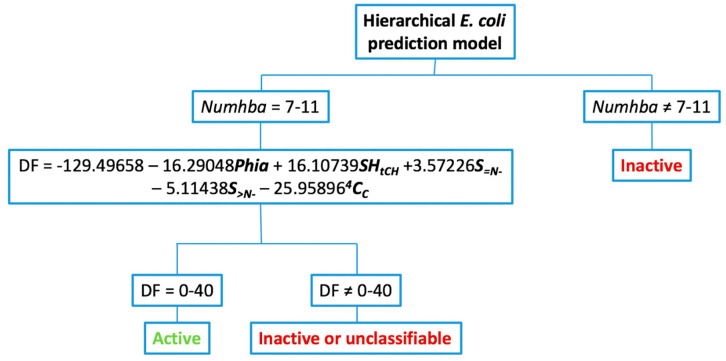
Decision tree for the application of the hierarchical model to predict antibacterial activity against *E. coli.*

Having established the maximum probability range for the discrete index *Numhba* and the corresponding DF, the hierarchical model was constructed (Figure 3).

### 2.2. Virtual Screening of Adapted DrugBank Database

Once the DrugBank database had been adapted, the model described above was applied to identify drugs with theoretical antibacterial activity against *E. coli*. The model selected 134 drugs as theoretically active (Table 2 and Supplementary Material, Table S7). Of these, 8 were already described as antibacterial drugs against *E. coli*, which provides an initial success rate of 6.1%. The remaining drugs can be classified as 67 marketed drugs for different pathologies and 55 drugs in experimental stages.

## 3. Discussion

Regarding the methodology used to develop de tree-based QSAR model, LDA is a frequently used method for the development of QSAR models [25,26,27,28], however, it has a key limitation: variables with discrete values cannot be used for the development of the model since they cannot be analyzed using the same statistical methods [29]. This is an important issue in the development of QSAR models because discrete descriptors contain relevant information that could be useful in predicting pharmacological activity. The formalism for the development of QSAR/QSPR models using LDA is based on the hypothesis that there is a group of compounds, (Ω), where each compound shall be denoted by ω∈Ω and the existence of a series of indexes we will design as ${\left\{I_{i}\right\}}_{i\in I}$, each element of this set is formed by the function I:Ω→ℝ, so that each compound has an assigned value for each index described [28]. If I(Ω) takes values in a discrete or numerable set, we face a discrete index. If, on the other hand, I(Ω) has at least an open interval of the real line, we face a continuous index. In this context, given any finite subset J⊂I, we can consider the finite subset ${\left\{I_{i}\right\}}_{i\in J}$ of indexes indexed by the subset J and obtain the values for each compound ω∈Ω as follows:

$$I_{J}\left(\omega \right)={\left(I_{j}\left(\omega \right)\right)}_{i\in J}.$$

To construct a linear discriminant function associated to the set of indexes ${\left\{I_{i}\right\}}_{i\in J}$, we have to endow the set of compounds with a spatial probability structure (Ω,F,ℙ). Thus, the linear discriminant function is constructed as:

(2) $$DF_{J}\left(x\right)=\mathbb{P}\left(G_{1}|I_{J}=x\right)-\mathbb{P}\left(G_{2}|I_{J}=x\right),$$

where

(3) $$\mathbb{P}\left(G_{i}|I_{J}=x\right)=\frac{\mathbb{P}(G_{i}\cap \left\{I_{J}=x\right\}}{\mathbb{P}\left(I_{J}=x\right)}=\frac{\mathbb{P}(I_{J}=x|G_{i})\mathbb{P}\left(G_{i}\right)}{\mathbb{P}\left(I_{J}=x|G_{1}\right)\mathbb{P}\left(G_{1}\right)+\mathbb{P}(I_{J}=x|G_{2})\mathbb{P}\left(G_{2}\right)}$$

For i=1 (active), 2 (inactive) is the conditioned probability of belonging to one of the two groups ($G_{i}$) when the index obtained using the set *J* ⊂ *I* is equal to x. Given a compound **ω**, we will calculate its indexes $I_{J}\left(\omega \right)=x\;$and will say that

(4) $$\omega \in \{\begin{array}{c} G_{1}\;\mathrm{if}\;DF_{J}\left(x\right)>0\\ G_{2}\;\mathrm{if}\;DF_{J}\left(x\right)<0 \end{array}$$

The usual hypotheses for the construction of $DF_{J}$ are:

$$\mathbb{P}\left(G_{1}\right)=\mathbb{P}(G_{2}).$$

$\mathbb{P}(I=x|G_{i})$ is a density function of multivariable normal distribution $N\left({µ}_{i}\left(J\right),\Sigma \left(J\right)\right)$ for i=1,2.

As a consequence of condition II, we can only assure the construction of the function $DF_{J}\left(x\right)$ if the indexes for $I_{j}$ when j∈J are continuous variables. Consequently, we must exclude all discrete variables if we want to use this statistical technique.

A natural question that appears is, what happens when we want to use a discrete index *I* that we cannot include in our index collection ${\left\{I_{j}\right\}}_{j\in J}$ for its use with LDA because it does not fulfill condition II. In order to solve this issue, we assume the following hypothesis:

We have an event Α :={a ≤I ≤b} and a natural number big enough $n_{0}\;>1$ in a way that

(5) $$\frac{\mathbb{P}(G_{1}|A)}{\mathbb{P}(G_{2}|A)}=\frac{\mathbb{P}(G_{1}|A)}{1-\mathbb{P}(G_{1}|A)}>n_{0},$$

that is, the probability of belonging to the active compounds group ($G_{1}$) when the discrete index has values within a fixed interval (satisfying property *A*) is higher than the probability of belonging to the inactive group ($G_{2}$). We will consider *A^c^* the opposite event of *A*, that is, when the discrete index has values outside the interval of maximum probability. In this case $A^{c}\;:=\left\{I\;<a\right\}\cup \{I>b\}$.

In particular, this is equal to the condition

(6) $$\mathbb{P}\left(G_{1}|A\right)>1-\frac{1}{n_{0}+1}.$$

The consequence we can infer from the latter expression and the fact that

(7) $$\mathbb{P}\left(G_{1}|A^{c}\right)=1-\mathbb{P}\left(G_{2}|A^{c}\right)\;y\;\mathbb{P}\left(G_{2}|A\right)=1-\mathbb{P}\left(G_{1}|A\right)$$

is

(8) $$\mathbb{P}\left(G_{1}|A\right)=\mathbb{P}\left(G_{1}|A^{c}\right)>1-\frac{1}{n_{0}+1}$$

This means that the probability of belonging to the inactive group ($G_{2}$) when the discrete index does not have values within the desired interval (does not satisfy *A*) is higher than the probability of belonging to the active group ($G_{1}$) under the same condition. This allows for the creation of a hierarchical model. We first construct two probability spaces (A*,* $F_{A}$*,* ${\mathbb{P}}_{A}$) and ($A^{c},\;F_{A^{c}},\;{\mathbb{P}}_{A^{c}}$) where

(9) $${\mathbb{P}}_{A}\left(B\right)≔\mathbb{P}\left(B|A\right)\;y\;{\mathbb{P}}_{A^{c}}\left(B\right)≔\mathbb{P}\left(B|A^{c}\right)$$

This allows us to construct, under the appropriate hypothesis, the prediction model we have named hierarchical:

If the molecule ω∈A, that is, it satisfies that a ≤I(ω) ≤b, we construct a discriminant linear function

(10) $$DF_{J}^{\left(A\right)}\left(x\right)={\mathbb{P}}_{A}(G_{1}|I_{J}=x)-{\mathbb{P}}_{A}\left(G_{2}|I_{J}=x\right)$$

If the molecule ω∉A, that is, it satisfies that I(ω)>b or that I(ω)<a, we construct the discriminant linear function

(11) $$DF_{J}^{\left(A^{c}\right)}\left(x\right)={\mathbb{P}}_{A^{c}}(G_{1}|I_{J}=x)-{\mathbb{P}}_{A^{c}}\left(G_{2}|I_{J}=x\right)$$

In conclusion, this model provides a hyperplane which separates two regions, allowing the discrimination between active and inactive compounds [30]. By incorporating the decision tree, a high probability sub-space is created which, implicitly, aims towards increasing the precision of the model as the molecules used to calculate de DF already belong to this space of higher probability of activity.

The model was constructed using a combined CART + LDA approach for several reasons. Firstly, the methodology used in the present paper was developed after identifying the impossibility of using discrete indexes with LDA. Additionally, the data available for many of the molecules used in the development of the model was limited to in vitro activity against *E. coli*. This means that there was no information regarding in vivo activity or ADMET properties, which immediately discarded the option of a mt-QSAR or MOOP-based QSAR model. Furthermore, the software that was available for the development of the model was limited to R and BMDP. Lastly, as it was mentioned in the introduction, this model was developed to identify drug candidates for repurposing, which implies that, for most cases, ADMET and other properties of the theoretically active compounds selected by the model are already known.

Focusing on the indexes selected by the model, the discrete index *Numhba*, which takes into account the number of hydrogen bond acceptors in the molecule, is interesting because the presence of a certain number of hydrogen bond acceptors in a molecule results in a better pharmacological profile when administered orally, which is especially interesting for quinolones [30]. This is one of the properties used in Lipinski’s “Rule of 5” in which *Numhba*, considering nitrogen and oxygen, must be ≤ 10, seeing as molecules with a higher number have many interactions with water and hinders transport through the lipid bilayer [31]. In this case, *Numhba* considers nitrogen, oxygen and fluorine as hydrogen bond acceptors.

As for the indexes selected for the DF, the electrotopological index for hydrogen atoms bonded to tertiary carbons (*SHtCH*) and the electrotopological index for imines (*S=N−*) have a positive effect on the DF value. On the other hand, the number of rotational bonds, represented by the *Phia* (ϕ) index, which refers to the flexibility of the molecule [32], the electrotopological index for tertiary nitrogens (*S>N−*) and the quotient between non-valence and valence order 4 cluster index (*4C_C_*) have a negative effect on the value of the DF.

The negative sign associated to the electrotopological index for tertiary nitrogens (*S>N−*), which indicated an unfavorable influence, is especially interesting. However, as the structure-activity relationship of quinolones states, this group in position 1 is an essential part of the pharmacophore [33]. Thus, we can deduce that it is an excess of this group what would affect activity negatively. Moreover, the value of this index is also influenced by the electronegative groups surrounding the tertiary nitrogen atoms [34].

The authors decided to use this model for the virtual screening of DrugBank because it is an open access database with information about more than 11,200 drugs which can be used for drug discovery and repurposing [24].

Many research [35,36,37,38,39,40,41,42,43,44,45,46] groups are taking similar approaches to repurpose drugs for which safety and toxicity data have been already collected from clinical assays in order to use them as antibiotic compounds with new pharmacological activity. (Table 3).

Regarding the drug candidates selected by the prediction model, besides the 8 antibacterial drugs mentioned previously, there were 10 antiviral and 12 antineoplastic compounds. These are especially interesting because both types of drugs are destined towards the destruction of undesired cells, which could include bacteria. In the same way that quinolones have both, antibacterial and cytotoxic activities [47], the mechanisms of action by which these 22 drugs inhibit or destroy viruses and tumor cells could also affect bacterial cells. In fact, recent studies suggest the possibility of reusing antineoplastic drugs for the treatment of bacterial infections [48,49]. Along these lines, Shah et al. have already described the antibacterial activity of antineoplastic drugs against drug-resistant *Escherichia coli* among other bacteria [50].

On the other hand, the model also selected 12 glucocorticoids. Any compound in this therapeutic group is not an interesting candidate when it comes to repositioning it as an antibacterial drug because they suppress the immune response by blocking antimicrobial autophagy and nitric oxide production [51,52]. This, in turn, could worsen symptoms and extend the duration of the bacterial infection. However, these molecules could be used as lead compounds and, via pharmacomodulation, reduce their immunosuppressive effect while improving their antibacterial activity [53].

## 4. Materials and Methods

### 4.1. Tree-Based QSAR Model Construction

The procedure to use the hierarchical methodology presented in the discussion was used to obtain a tree-based prediction method following the steps presented below.

*1.*  Compound selection and index calculation: the compounds selected to build the prediction model must belong to the same structural family. These compounds were divided into two groups, active and inactive. Once the group of compounds had been selected, the molecular descriptors or indexes of each of the molecules were calculated using MOLCONN-Z [54] and DESMOL13 [55] software.
*2.*  Discrete index analysis: the discrete indexes were analyzed to determine if there were any with a value range that grouped the active compounds. The condition of a compound having the value of that discrete index within the range of maximum probability acted as the first step in the decision tree. Furthermore, this analysis could also act as a fast way to determine new structure activity relationships. It must be noted that, for indexes in which not enough inactive compounds are found in the higher probability space, this methodology cannot be applied for two reasons. Firstly, the lack of inactive compounds makes it impossible to obtain a discriminant function. Moreover, this type of index would lead to overfitting.
*3.*  Probability space construction: the compounds used for the construction of the model were separated in groups according to their value for the chosen discrete indexes.
*4.*  Linear Discriminant Analysis: a discriminant function was calculated for the selected group of molecules using only continuous indexes. This DF was calculated using the BMDP (BioMedicine Department Program) module 7M [56]. Compounds used to calculate the DF were randomly split into training and test groups by the BMDP software.
*5.*  Hierarchical decision tree construction: once the probability spaces of the discrete indexes had been determined and the corresponding discriminant functions (DFs) calculated, a hierarchical tree was built. In the first level of this decision tree, compounds were grouped according to their value for the selected discrete index. Those that lie outside of the maximum probability space were directly classified as inactive. The remaining compounds went on to the second level of the tree. In this level, the DF is applied. Compounds within the established highest activity expectancy range are classified as active, while the rest are classified as inactive or unclassifiable.

### 4.2. DrugBank Database Construction

In order to build the database from DrugBank, the structures were retrieved from ChemSpider, an open access database that allows easy access to more than 67 million chemical structures, properties and associated information. This database integrates compounds from hundreds of high quality databases among which is DrugBank. Moreover, ChemSpider allows the selection of one or several databases and the individual download of structures in “mol” format, numbered by default with the identification number from ChemSpider, which allows us to know which structures are included in our database.

Once the structures were downloaded, a series of modifications had to be carried out to transform them into chemical graphs that our index calculation softwares, MOLCONN-Z and DESMOL11, could use. All the modifications explained below were made using the drawing software ChemDraw Professional 17.0 from the ChemOffice 2017 software package.

Firstly, the stereochemistry had to be removed from all molecules, seeing as the index calculation softwares only work with the 2D graph. This had to be done one molecule at a time, changing the bonds that indicated chirality for flat bonds. Furthermore, these softwares do not accept molecules with more than 99 bonds from end to end of the molecule thus, compounds exceeding 99 bonds had to be removed. Moreover, we were only able to calculate the indexes of molecules containing C, F, Cl, Br, I, O, N, S, P, B, Si, Ge, Sn and Pb, as well as hydrogen, thus, each molecule had to be analyzed to detect any other atoms and delete them. Having taken all these considerations, we were able to build our own DrugBank subset with 7031 chemical graphs of approved and experimental drugs.

## 5. Conclusions

The prediction QSAR model constructed using a hierarchical tree-based methodology has selected 134 drugs with theoretical antibacterial activity against *E. coli*. Of these, 22 could be interesting candidates to be further studied due to their proven pharmacological activity, which could provide new mechanisms of action, further contributing to combat emerging antibiotic resistance. As a result, drug repurposing appears as a cost- and time-effective alternative for the development of new antibacterial drugs, broadening the existing pipeline. Furthermore, the topological indexes *Nclass* and *Numhba* have proven to have the ability to group active compounds effectively, which suggests a close relationship between them and the antibacterial activity of compounds against *E. coli*.

## Figures and Tables

**Table 1:** Classification matrix for DF.

Group	Active	Inactive	% Hits
Active *training*	28	0	100
Inactive *training*	0	8	100
Active *test*	11	0	100
Inactive *test*	0	1	100
TOTAL	39	9	100

**Table 2:** Summary of theoretically active compounds against *E. coli* selected by the model.

Therapeutic Use	Nº of Selected Candidates
Antineoplastic	12
Glucocorticoid	12
Antiviral	10
Antibiotic	8
Anti-inflammatory	4
Antioxidant	3
Neuroprotector	3
Bronchodilator	2
Diabetic neuropathy	2
Glutamate receptor	2
Growth factor	2
Immunomodulator	2
Alzheimer	1
Anti-convulsive	1
Antifungal	1
Anti-infective	1
Anti-rheumatic	1
Antitussive	1
Anxiolytic	1
Benzodiazepine antagonist	1
Cardiotonic	1
Diabetes	1
GABA antagonist	1
Mannosidase inhibitor	1
Nucleoside	1
Phenylketonuria	1
Protein	1
Pulmonary arterial hypertension	1
Vitamin	1
Experimental drugs	55

**Table 3:** Drug candidates to be repurposed with in vitro antibacterial activity.

Drug	Therapeutic Use	Antibacterial Activity	Ref.
Loperamide	Antidiarrheal	*Salmonella enterica*	[35]
Auranofin	Rheumatoid arthritis	MRSA	[36]
Ebselen	No clinical use	MRSA	[37]
Ivermectin	Anthelmintic	*M. tuberculosis*	[38]
Entacapone	Anti-Parkinson	*M. tuberculosis*	[39]
Thioridazine	Antipsychotic	*M. tuberculosis*	[40]
5-Fluorouracil	Antineoplastic	Broad spectrum	[41]
Niclosamide	Anthelmintic	*P. aeruginosa*	[42]
Diflunisal	Anti-inflammatory	MRSA	[43]
Statins	Hypolipidemic	*P. aeruginosa*	[44]
Terfenadine	Antihistaminic	*S. aureus*	[45]
		*M. tuberculosis*	
Zafirlukast	Asthma	*M. tuberculosis*	[46]

## Supplementary Materials

The following are available online at https://www.mdpi.com/1424-8247/13/12/431/s1, Table S1: Active compounds against *E. coli*, Table S2: Inactive compounds against *E. coli*, Table S3: Results obtained in the LDA and compounds classification according to the DF, active training group, Table S4: Results obtained in the LDA and compound classification according to the DF, inactive training group, Table S5: Results obtained in the LDA and compounds classification according to the DF, active test group, Table S6: Results obtained in the LDA and compound classification according to the DF, inactive test group, Table S7: Theoretically active compounds against *E. coli* selected by the model, Figure S1: Probability distribution diagrams of the analyzed discrete indexes.
