# Supplementary material for: Tree-Based QSAR Model for Drug Repurposing in the Discovery of New Antibacterial Compounds against Escherichia coli
Source: Pharmaceuticals (Basel). 2020 Nov 28;13(12):431. doi: 10.3390/ph13120431 (PMC7760995; doi:10.3390/ph13120431)
Supplement: Supplementary file 1 [file pharmaceuticals-13-00431-s001.zip › Supplementary Material B Suay-Garcia/Supp Info 1 Table 1S-2S.pdf]

Table 1S. Active compounds against *E. coli*

Compound	IUPAC Name	Structure	Reference
Act1	(*E*)-7-(3-amino-4-(methoxyimino)piperidin-1-yl)-6-fluoro-1-(2-fluorocyclopropyl)-8-methoxy-4-oxo-1,4-dihydroquinoline-3-carboxylic acid	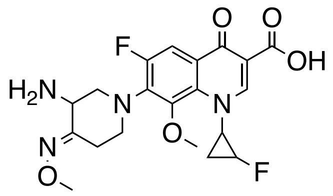	Liu H, Huang J, Wang J et al. Synthesis, antimycobacterial and antibacterial evaluation of I-[(1R, 2S)-2-fluorocyclopropyl]fluoroquinolone derivatives containing an oxime functional moiety. Eur J Med Chem 2014; 86:628-638.
Act2	(*E*)-7-(2-(aminomethyl)-3-(methoxyimino)azetidin-1-yl)-6-fluoro-1-(2-fluorocyclopropyl)-8-methoxy-4-oxo-1,4-dihydroquinoline-3-carboxylic acid	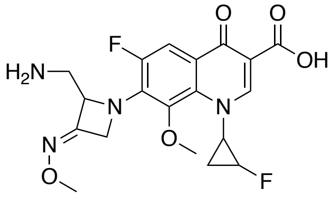	Idem
Act3	(*E*)-7-(3-amino-4-(methoxyimino)piperidin-1-yl)-6-fluoro-1-(2-fluorocyclopropyl)-4-oxo-1,4-dihydroquinoline-3-carboxylic acid	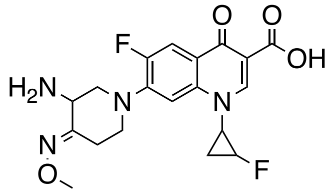	Idem
Act4	(*Z*)-7-(3-(aminomethyl)-4-(methoxyimino)pyrrolidin-1-yl)-6-fluoro-1-(2-fluorocyclopropyl)-4-oxo-1,4-dihydroquinoline-3-carboxylic acid	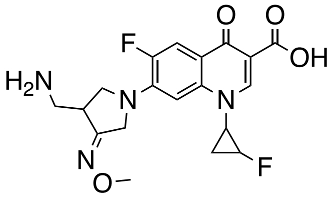	Idem
Act5	(*Z*)-7-(3-(aminomethyl)-4-(ethoxyimino)pyrrolidin-1-yl)-6-fluoro-1-(2-fluorocyclopropyl)-4-oxo-1,4-dihydroquinoline-3-carboxylic acid	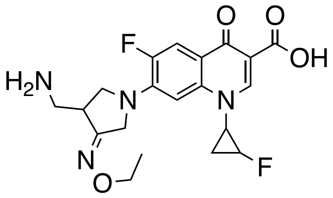	Idem
Act6	(*Z*)-7-(3-(aminomethyl)-4-(phenoxyimino)pyrrolidin-1-yl)-6-fluoro-1-(2-fluorocyclopropyl)	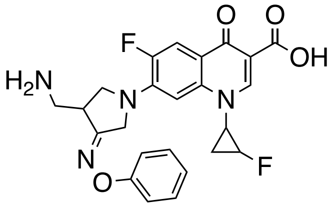	Idem

	-4-oxo-1,4-dihydroquinoline-3-carboxylic acid		
Act7	(*E*)-7-(2-(aminomethyl)-3-(methoxyimino)azetidin-1-yl)-6-fluoro-1-(2-fluorocyclopropyl)-4-oxo-1,4-dihydroquinoline-3-carboxylic acid	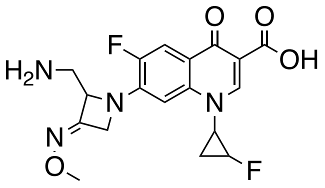	Idem
Act8	(*E*)-7-(2-(aminomethyl)-3-(ethoxyimino)azetidin-1-yl)-6-fluoro-1-(2-fluorocyclopropyl)-4-oxo-1,4-dihydroquinoline-3-carboxylic acid	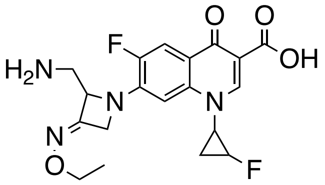	Idem
Act9	(*E*)-7-(2-(aminomethyl)-3-(phenoxyimino)azetidin-1-yl)-6-fluoro-1-(2-fluorocyclopropyl)-4-oxo-1,4-dihydroquinoline-3-carboxylic acid	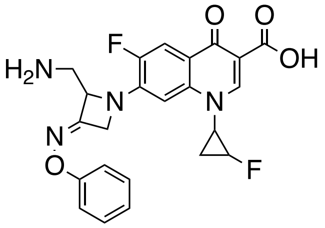	Idem
Act13	(*Z*)-7-(3-(aminomethyl)-4-(methoxyimino)pyrrolidin-1-yl)-8-chloro-6-fluoro-1-(2-fluorocyclopropyl)-4-oxo-1,4-dihydroquinoline-3-carboxylic acid	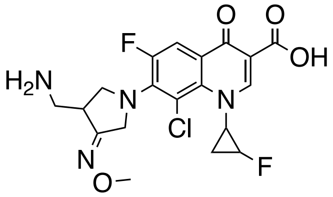	Idem
Act14	(*Z*)-7-(3-(aminomethyl)-4-(ethoxyimino)pyrrolidin-1-yl)-8-chloro-6-fluoro-1-(2-fluorocyclopropyl)-4-oxo-1,4-dihydroquinoline-3-carboxylic acid	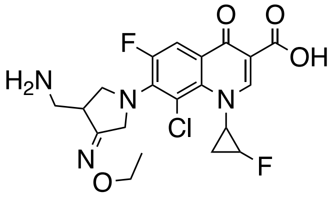	Idem
Act15	(*Z*)-7-(3-(aminomethyl)-4-(phenoxyimino)pyrrolidin-1-yl)-8-chloro-6-fluoro-1-(2-fluorocyclopropyl)	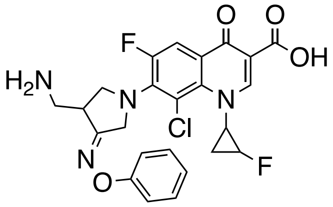	Idem

	-4-oxo-1,4-dihydroquinoline-3-carboxylic acid		
Act16	(*E*)-7-(3-amino-4-(methoxyimino)piperidin-1-yl)-6-fluoro-1-(2-fluorocyclopropyl)-4-oxo-1,4-dihydro-1,8-naphthyridine-3-carboxylic acid	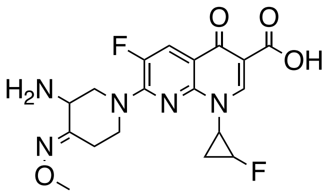	Idem
Act17	(*Z*)-7-(3-(aminomethyl)-4-(methoxyimino)pyrrolidin-1-yl)-6-fluoro-1-(2-fluorocyclopropyl)-4-oxo-1,4-dihydro-1,8-naphthyridine-3-carboxylic acid	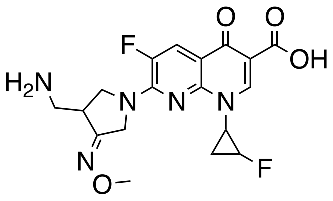	Idem
Act18	(*Z*)-7-(3-(aminomethyl)-4-(ethoxyimino)pyrrolidin-1-yl)-6-fluoro-1-(2-fluorocyclopropyl)-4-oxo-1,4-dihydro-1,8-naphthyridine-3-carboxylic acid	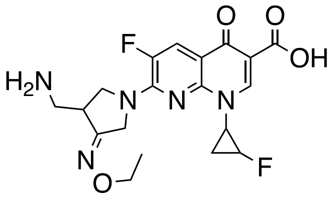	Idem
Act19	(*Z*)-7-(3-(aminomethyl)-4-(phenoxyimino)pyrrolidin-1-yl)-6-fluoro-1-(2-fluorocyclopropyl)-4-oxo-1,4-dihydro-1,8-naphthyridine-3-carboxylic acid	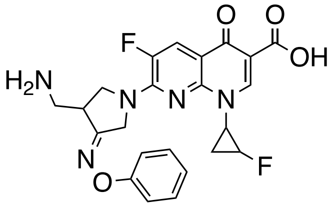	Idem
Act20	(*E*)-7-(2-(aminomethyl)-3-(methoxyimino)azetidin-1-yl)-6-fluoro-1-(2-fluorocyclopropyl)-4-oxo-1,4-dihydro-1,8-naphthyridine-3-carboxylic acid	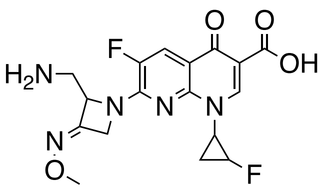	Idem
Act21	(*E*)-7-(2-(aminomethyl)-3-(ethoxyimino)azetidin-1-yl)-6-fluoro-1-(2-	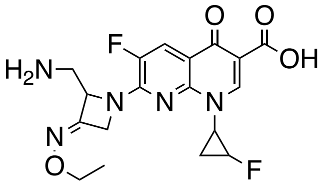	Idem

	fluorocyclopropyl)-4-oxo-1,4-dihydro-1,8-naphthyridine-3-carboxylic acid		
Act22	(*E*)-7-(2-(aminomethyl)-3-(phenoxyimino)azetidin-1-yl)-6-fluoro-1-(2-fluorocyclopropyl)-4-oxo-1,4-dihydro-1,8-naphthyridine-3-carboxylic acid	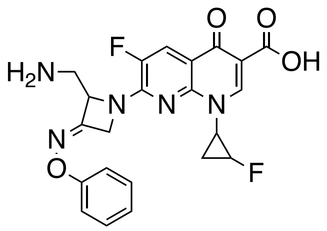	Idem
Act26	6,8-difluoro-1-(4-fluorophenyl)-7-(4-(2-((4-(2-fluorophenyl)-5-oxo-2,5-dihydrofuran-3-yl)oxy)ethyl)piperazin-1-yl)-4-oxo-1,4-dihydroquinoline-3-carboxylic acid	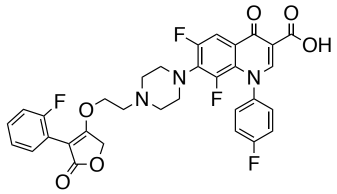	Wang XD, Wei W, Wang PF et al. Novel 3-arylfuran-2(5H)-one-fluoroquinolone hybrid: design, synthesis and evaluation as antibacterial agent. Bioorg Med Chem 2014, 22(14):3620-3628.
Act27	1-cyclopropyl-6,8-difluoro-7-(4-(2-((4-(2-fluorophenyl)-5-oxo-2,5-dihydrofuran-3-yl)oxy)ethyl)piperazin-1-yl)-4-oxo-1,4-dihydroquinoline-3-carboxylic acid	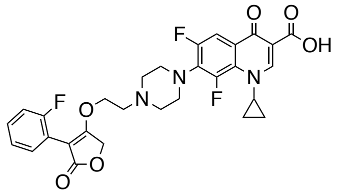	Wang XD, Wei W, Wang PF et al. Novel 3-arylfuran-2(5H)-one-fluoroquinolone hybrid: design, synthesis and evaluation as antibacterial agent. Bioorg Med Chem 2014, 22(14):3620-3628.
Act32	(*Z*)-7-(3-(aminomethyl)-4-(methoxyimino)-3-methylpiperidin-1-yl)-1-cyclopropyl-6,8-difluoro-4-oxo-1,4-dihydroquinoline-3-carboxylic acid	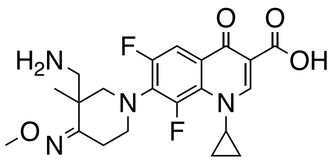	Chai Y, Liu M, Wang B, et al. Synthesis and in vitro antibacterial activity of novel fluoroquinolone derivatives containing substituted piperidines. Bioorg Med Chem Lett 2010; 20(17):5195-5198.
Besifloxacin	7-(3-aminoazepan-1-yl)-8-chloro-1-cyclopropyl-6-fluoro-4-oxo-1,4-dihydroquinoline-3-carboxylic acid	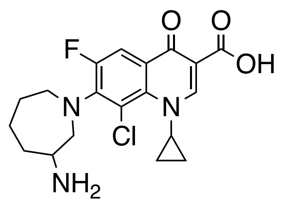	CLSI Performance Standards for Antimicrobial Susceptibility Testing, 26th Edition.

Ciprofloxacin	1-cyclopropyl-6-fluoro-4-oxo-7-(piperazin-1-yl)-1,4-dihydroquinoline-3-carboxylic acid	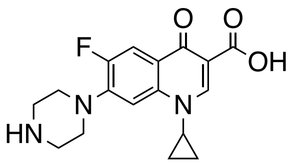	Idem
Clinafloxacin	7-(3-aminopyrrolidin-1-yl)-8-chloro-1-cyclopropyl-6-fluoro-4-oxo-1,4-dihydroquinoline-3-carboxylic acid	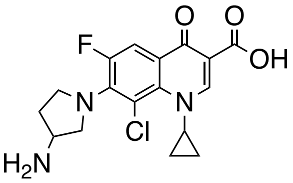	Idem
Delafloxacin	1-(6-amino-3,5-difluoropyridin-2-yl)-8-chloro-6-fluoro-7-(3-hydroxyazetidin-1-yl)-4-oxo-1,4-dihydroquinoline-3-carboxylic acid	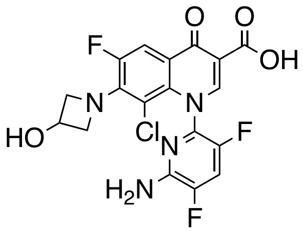	Idem
Enoxacin	1-ethyl-6-fluoro-4-oxo-7-(piperazin-1-yl)-1,4-dihydro-1,8-naphthyridine-3-carboxylic acid	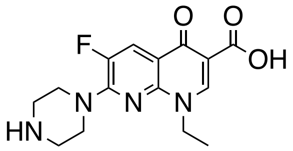	Idem
Finafloxacin	8-cyano-1-cyclopropyl-6-fluoro-7-(hexahydropyrrolo[3,4-*b*][1,4]oxazin-6(2*H*)-yl)-4-oxo-1,4-dihydroquinoline-3-carboxylic acid	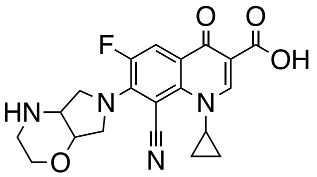	Idem
Fleroxacin	6,8-difluoro-1-(2-fluoroethyl)-7-(4-methylpiperazin-1-yl)-4-oxo-1,4-dihydroquinoline-3-carboxylic acid	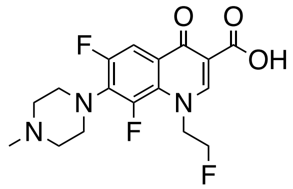	Idem
Garenoxacin	1-cyclopropyl-8-(difluoromethoxy)-6-fluoro-7-(1-methylisoindolin-5-yl)-4-oxo-1,4-dihydroquinoline-3-carboxylic acid	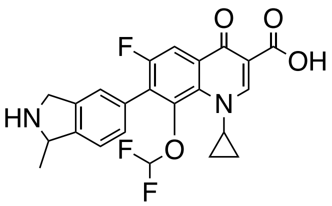	Idem
Gatifloxacin	1-cyclopropyl-6-fluoro-8-methoxy-7-(3-methylpiperazin-1-yl)-4-oxo-1,4-dihydroquinoline-3-carboxylic acid	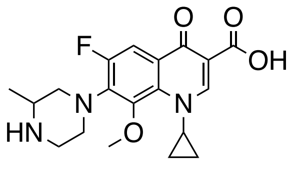	Idem

Gemifloxacin	(*Z*)-7-(3-(aminomethyl)-4-(methoxyimino)pyrrolidin-1-yl)-1-cyclopropyl-6-fluoro-4-oxo-1,4-dihydro-1,8-naphthyridine-3-carboxylic acid	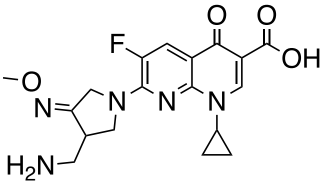	Idem
Grepafloxacin	1-cyclopropyl-6-fluoro-5-methyl-7-(3-methylpiperazin-1-yl)-4-oxo-1,4-dihydroquinoline-3-carboxylic acid	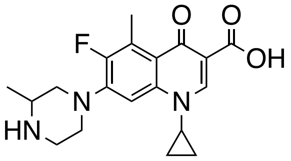	Idem
Levofloxacin	9-fluoro-3-methyl-10-(4-methylpiperazin-1-yl)-7-oxo-2,3-dihydro-7*H*-[1,4]oxazino[2,3,4-*ij*]quinoline-6-carboxylic acid	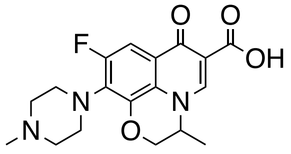	Idem
Lomefloxacin	1-ethyl-6,8-difluoro-7-(3-methylpiperazin-1-yl)-4-oxo-1,4-dihydroquinoline-3-carboxylic acid	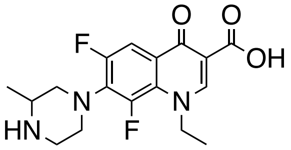	Idem
Moxifloxacin	1-cyclopropyl-6-fluoro-8-methoxy-7-(octahydro-6*H*-pyrrolo[3,4-*b*]pyridin-6-yl)-4-oxo-1,4-dihydroquinoline-3-carboxylic acid	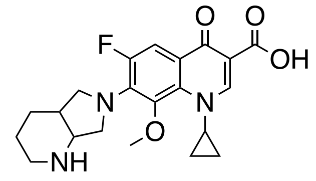	Idem
Norfloxacin	1-ethyl-6-fluoro-4-oxo-7-(piperazin-1-yl)-1,4-dihydroquinoline-3-carboxylic acid	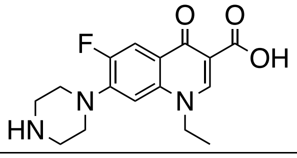	Idem
Ofloxacin	9-fluoro-3-methyl-10-(4-methylpiperazin-1-yl)-7-oxo-2,3-dihydro-7*H*-[1,4]oxazino[2,3,4-*ij*]quinoline-6-carboxylic acid	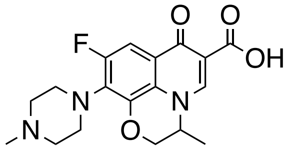	Idem
Pradofloxacin	8-cyano-1-cyclopropyl-6-fluoro-7-(octahydro-6*H*-pyrrolo[3,4-*b*]pyridin-6-yl)-4-	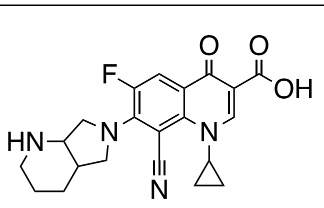	Pridmore A, Stephan B, Griefe HA. In vitro activity of pradofloxacin against clinical isolates from European field studies In: Proceedings

	oxo-1,4-dihydroquinoline-3-carboxylic acid		of the American Society for Microbiology (ASM) 105th General Meeting, Atlanta, GA, USA p617, 2005.<br><br>Abraham J, Ewet K, de Jong A. Pradofloxacin: Comparative in vitro activity against selected pathogens from the US. In: 42nd Interscience Conference on Antimicrobial Agents and Chemotherapy, San Diego, CA, USA p.189.
Sparfloxacin	5-amino-1-cyclopropyl-7-(3,5-dimethylpiperazin-1-yl)-6,8-difluoro-4-oxo-1,4-dihydroquinoline-3-carboxylic acid	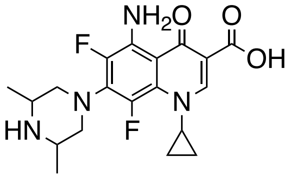	Idem
Trovafloxacin	7-(6-amino-3-azabicyclo[3.1.0]hexan-3-yl)-1-(2,4-difluorophenyl)-6-fluoro-4-oxo-1,4-dihydro-1,8-naphthyridine-3-carboxylic acid	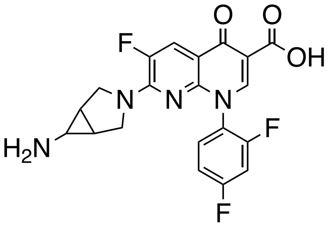	Idem
Ulifloxacin	6-fluoro-1-methyl-4-oxo-7-(piperazin-1-yl)-2a,3-dihydro-1*H*,4*H*-[1,3]thiazeto[3,2-*a*]quinoline-3-carboxylic acid	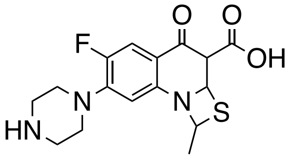	Idem
PGE-8367769	1-cyclopropyl-6-fluoro-7,8-dimethyl-4-oxo-1,4-dihydroquinoline-3-carboxylic acid	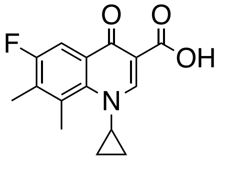	Macinga DR, Renick PJ, Makin KM, et al. Unique biological properties and molecular mechanism of 5,6-bridged quinolones. Antimicrob Agents Chemother. 2003; 47(8):2526-2537.

Table 2S. Inactive compounds against *E. coli*

Compound	IUPAC Name	Structure	Reference
Inact1	1-ethyl-6,8-difluoro-4-oxo-7-(4-(((3,4,5-triacetoxy-6-(acetoxymethyl)tetrahydro-2*H*-pyran-2-yl)oxy)methyl)-1*H*-1,2,3-triazol-1-yl)-1,4-dihydroquinoline-3-carboxylic acid	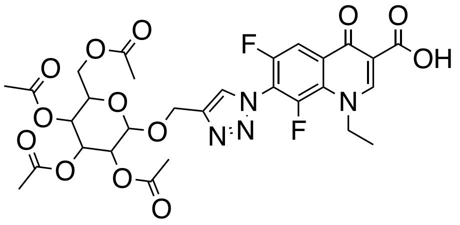	Azad CS, Bhunia SS, Krishna A, Shukla PK, Saxena AK. Novel Glycoconjugate of 8-Fluoro Norfloxacin Derivatives as Gentamicin-resistant Staphylococcus aureus Inhibitors: Synthesis and Molecular Modelling Studies. Chem Biol Drug Des. 2015; 86(4):440-446.
Inact2	7-(4-(((3-acetamido-4,5-diacetoxy-6-(acetoxymethyl)tetrahydro-2*H*-pyran-2-yl)oxy)methyl)-1*H*-1,2,3-triazol-1-yl)-1-ethyl-6,8-difluoro-4-oxo-1,4-dihydroquinoline-3-carboxylic acid	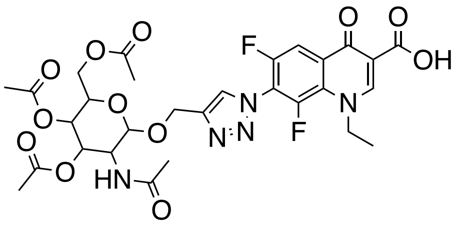	Idem
Inact3	1-ethyl-6,8-difluoro-4-oxo-7-(4-(2-((5-oxo-4-phenyl-2,5-dihydrofuran-3-yl)oxy)ethyl)piperazin-1-yl)-1,4-dihydroquinoline-3-carboxylic acid	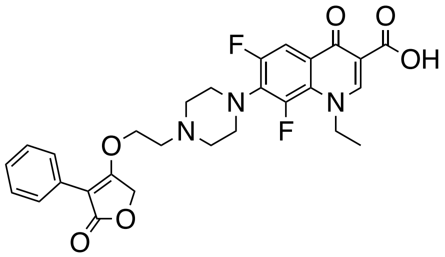	Wang XD, Wei W, Wang PF et al. Novel 3-arylfuran-2(5H)-one-fluoroquinolone hybrid: design, synthesis and evaluation as antibacterial agent. Bioorg Med Chem. 2014, 22(14):3620-3628.
Inact4	7-(4-(2-((4-(4-chlorophenyl)-5-oxo-2,5-dihydrofuran-3-yl)oxy)ethyl)piperazin-1-yl)-1-ethyl-6,8-difluoro-4-oxo-1,4-dihydroquinoline-3-carboxylic acid	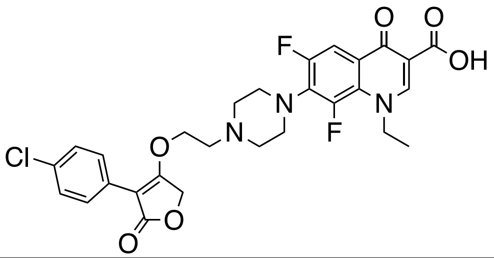	Idem

Inact5	1-cyclopropyl-6,8-difluoro-7-(4-(2-((4-(4-methoxyphenyl)-5-oxo-2,5-dihydrofuran-3-yl)oxy)ethyl)piperazin-1-yl)-4-oxo-1,4-dihydroquinoline-3-carboxylic acid	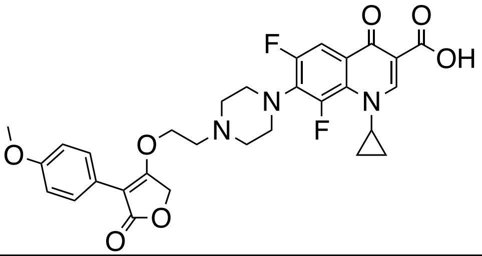	Idem
Inact6	1-cyclopropyl-6-fluoro-7-(4-(2-(4-methylpiperazin-1-yl)acetyl)piperazin-1-yl)-4-oxo-1,4-dihydroquinoline-3-carboxylic acid	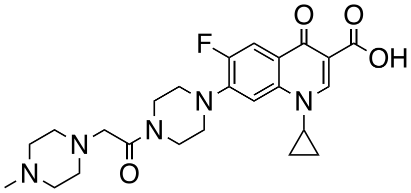	Suresh N, Nagesh HN, Renuka J, et al. Synthesis and evaluation of 1-cyclopropyl-6-fluoro-1,4-dihydro-4-oxo-7-(4-(2-(4-substitutedpiperazin-1-yl)acetyl)piperazin-1-yl)quinoline-3-carboxylic acid derivatices as anti-tubercular and antibacterial agents. Eur J Med. 2014; 71:324-332.
Inact7	1-cyclopropyl-7-(4-(2-(4-(3,4-difluorophenyl)piperazin-1-yl)acetyl)piperazin-1-yl)-6-fluoro-4-oxo-1,4-dihydroquinoline-3-carboxylic acid	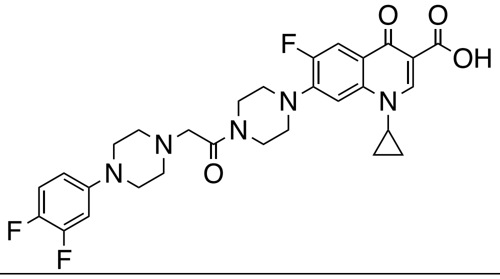	Idem
Inact8	7-(4-(2-(4-(bis(4-fluorophenyl)methyl)piperazin-1-yl)acetyl)piperazin-1-yl)-1-cyclopropyl-6-fluoro-4-oxo-1,4-dihydroquinoline-3-carboxylic acid	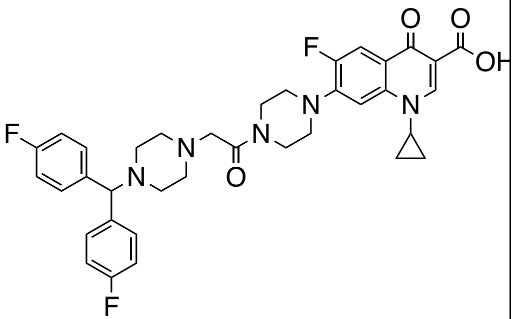	Idem
Inact9	1-ethyl-6-fluoro-7-(4-(5-(2-nitrophenyl)-1,3,4-thiadiazol-2-yl)piperazin-1-yl)-4-oxo-1,4-dihydroquinoline-3-carboxylic acid	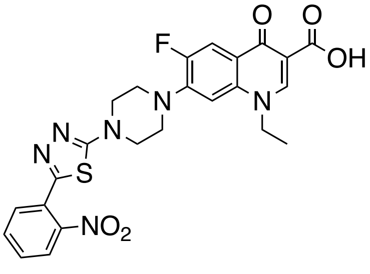	Foroumadi A, Soltani F, Moshafi MH, Ashraf-Askari R. Synthesis and in vitro antibacterial activity of some N-(5-aryl-1,3,4-thiadiazole-2-yl)piperazinyl quinolone derivatives.

			Farmaco. 2003; 58(10):1023-1028.
Inact10	1-ethyl-6-fluoro-7-(4-(5-(2-nitrophenyl)-1,3,4-thiadiazol-2-yl)piperazin-1-yl)-4-oxo-1,4-dihydro-1,8-naphthyridine-3-carboxylic acid	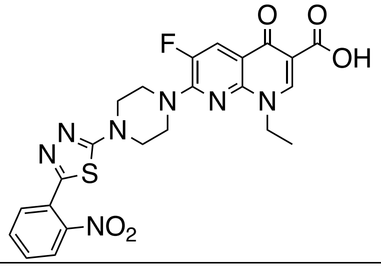	Idem
Inact11	1-cyclopropyl-6-fluoro-7-(4-(5-(3-nitrophenyl)-1,3,4-thiadiazol-2-yl)piperazin-1-yl)-4-oxo-1,4-dihydroquinoline-3-carboxylic acid	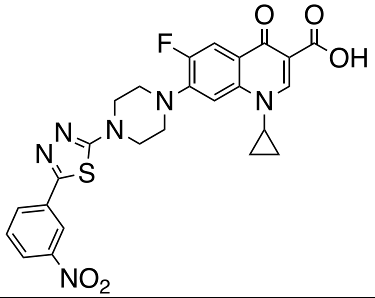	Idem
Inact12	1-ethyl-6-fluoro-7-(4-(5-(3-nitrophenyl)-1,3,4-thiadiazol-2-yl)piperazin-1-yl)-4-oxo-1,4-dihydroquinoline-3-carboxylic acid	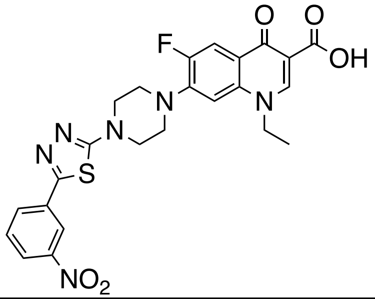	Idem
Inact13	1-ethyl-6-fluoro-7-(4-(5-(3-nitrophenyl)-1,3,4-thiadiazol-2-yl)piperazin-1-yl)-4-oxo-1,4-dihydro-1,8-naphthyridine-3-carboxylic acid	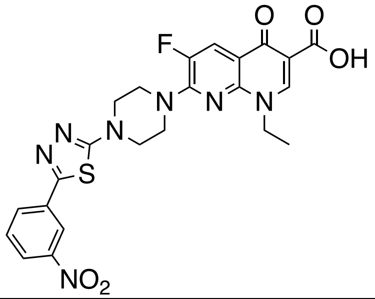	Idem
Inact14	1-cyclopropyl-6-fluoro-7-(4-(5-(4-nitrophenyl)-1,3,4-thiadiazol-2-yl)piperazin-1-yl)-4-oxo-1,4-dihydroquinoline-3-carboxylic acid	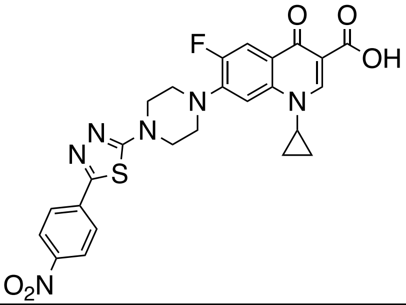	Idem
Inact15	1-ethyl-6-fluoro-7-(4-(5-(4-nitrophenyl)-1,3,4-thiadiazol-2-yl)piperazin-1-yl)-4-oxo-1,4-dihydroquinoline-3-carboxylic acid	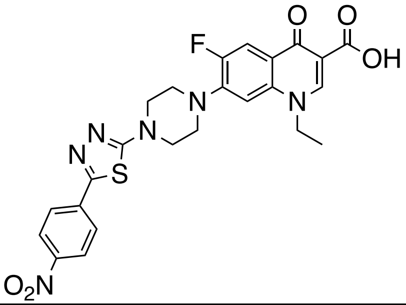	Idem

Inact16	1-ethyl-6-fluoro-7-(4-(5-(4-nitrophenyl)-1,3,4-thiadiazol-2-yl)piperazin-1-yl)-4-oxo-1,4-dihydro-1,8-naphthyridine-3-carboxylic acid	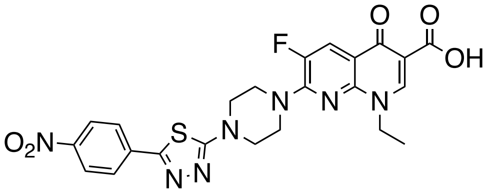	Idem
Inact17	1-cyclopropyl-6-fluoro-7-(4-(2-((6-nitrobenzo[*d*]thiazol-2-yl)amino)-2-oxoethyl)piperazin-1-yl)-4-oxo-1,4-dihydroquinoline-3-carboxylic acid	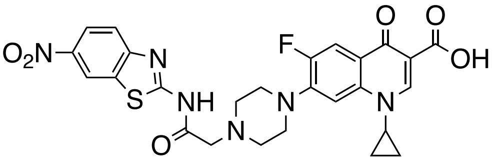	Sharma PC, Kumar R, Chaudhary M, Sharma A, Rajak H. Synthesis and biological evaluation of novel benzothiazole clubbed fluoroquinolone derivatives. J Enzyme Inhib Med Chem. 2013; 28(1):1-10.
Inact18	1-cyclopropyl-6-fluoro-7-(4-(2-((6-methylbenzo[*d*]thiazol-2-yl)amino)-2-oxoethyl)piperazin-1-yl)-4-oxo-1,4-dihydroquinoline-3-carboxylic acid	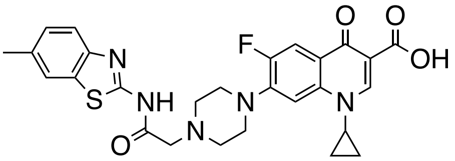	Idem
Inact19	1-cyclopropyl-6-fluoro-7-(4-(2-((6-fluorobenzo[*d*]thiazol-2-yl)amino)-2-oxoethyl)piperazin-1-yl)-4-oxo-1,4-dihydroquinoline-3-carboxylic acid	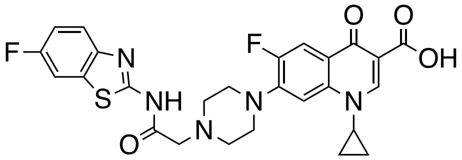	Idem
Inact20	7-(4-(2-((6-chlorobenzo[*d*]thiazol-2-yl)amino)-2-oxoethyl)piperazin-1-yl)-1-ethyl-6-fluoro-4-oxo-1,4-dihydroquinoline-3-carboxylic acid	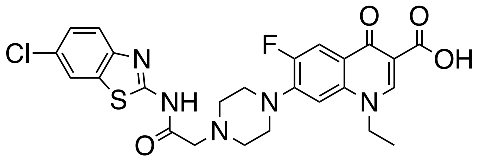	Idem
Inact21	7-(4-(2-((5,7-dichlorobenzo[*d*]thiazol-2-yl)amino)-2-oxoethyl)piperazin-1-yl)-1-ethyl-6-fluoro-4-oxo-1,4-dihydroquinoline-3-carboxylic acid	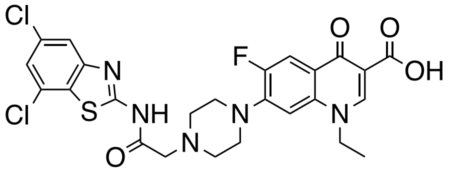	Idem
Inact22	1-ethyl-6-fluoro-7-(4-(2-((6-nitrobenzo[*d*]thiazol-2-yl)amino)-2-oxoethyl)piperazin-1-yl)-4-oxo-1,4-dihydroquinoline-3-carboxylic acid	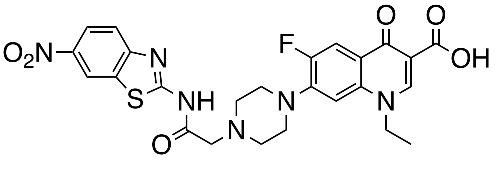	Idem

Inact23	1-ethyl-6-fluoro-7-(4-(2-((4-methylbenzo[*d*]thiazol-2-yl)amino)-2-oxoethyl)piperazin-1-yl)-4-oxo-1,4-dihydroquinoline-3-carboxylic acid	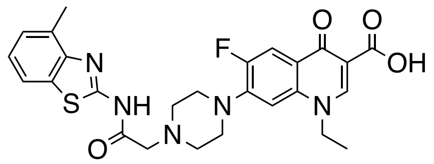	Idem
Inact24	1-ethyl-6-fluoro-7-(4-(2-((4-fluorobenzo[*d*]thiazol-2-yl)amino)-2-oxoethyl)piperazin-1-yl)-4-oxo-1,4-dihydroquinoline-3-carboxylic acid	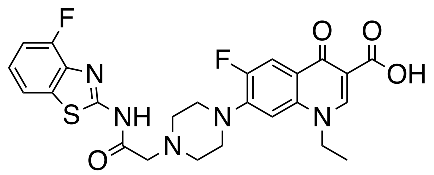	Idem
Inact25	7-(4-(2-((4-chlorobenzo[*d*]thiazol-2-yl)amino)-2-oxoethyl)-3-methylpiperazin-1-yl)-1-cyclopropyl-6-fluoro-8-methoxy-4-oxo-1,4-dihydroquinoline-3-carboxylic acid	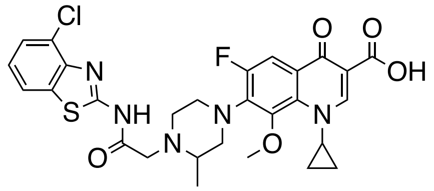	Idem
Inact26	1-cyclopropyl-7-(4-(2-((6,7-dichlorobenzo[*d*]thiazol-2-yl)amino)-2-oxoethyl)-3-methylpiperazin-1-yl)-6-fluoro-8-methoxy-4-oxo-1,4-dihydroquinoline-3-carboxylic acid	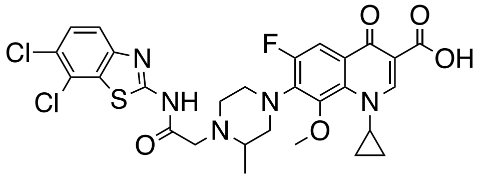	Idem
Inact27	1-cyclopropyl-6-fluoro-8-methoxy-7-(3-methyl-4-(2-((4-nitrobenzo[*d*]thiazol-2-yl)amino)-2-oxoethyl)piperazin-1-yl)-4-oxo-1,4-dihydroquinoline-3-carboxylic acid	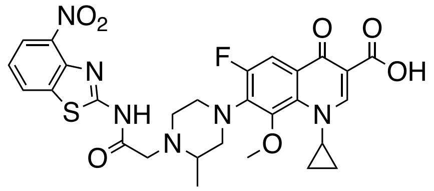	Idem
Inact28	1-cyclopropyl-6-fluoro-8-methoxy-7-(3-methyl-4-(2-((4-methylbenzo[*d*]thiazol-2-yl)amino)-2-oxoethyl)piperazin-1-yl)-4-oxo-1,4-dihydroquinoline-3-carboxylic acid	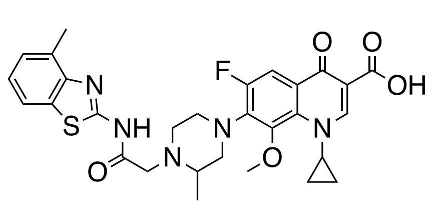	Idem
Inact29	1-cyclopropyl-6-fluoro-7-(4-(2-((4-fluorobenzo[*d*]thiazol-2-yl)amino)-2-oxoethyl)-3-methylpiperazin-1-yl)-8-methoxy-4-oxo-1,4-dihydroquinoline-3-carboxylic acid	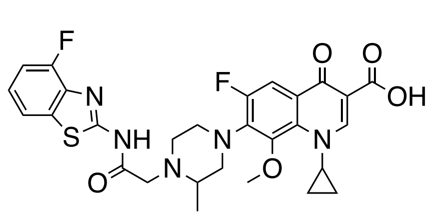	Idem

Inact30	(*Z*)-7-(3-(aminomethyl)-4-(ethoxyimino)-3-methylpiperidin-1-yl)-1-ethyl-6,8-difluoro-4-oxo-1,4-dihydroquinoline-3-carboxylic acid	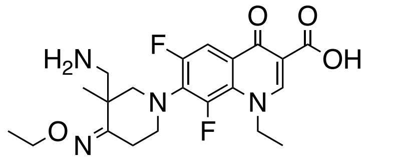	Chai Y, Liu M, Wang B, et al. Synthesis and in vitro antibacterial activity of novel fluoroquinolone derivatives containing substituted piperidines. Bioorg Med Chem Lett. 2010; 20(17):5195-5198.
Inact31	(*Z*)-7-(3-(aminomethyl)-4-(ethoxyimino)-3-methylpiperidin-1-yl)-6,8-difluoro-1-(2-fluoroethyl)-4-oxo-1,4-dihydroquinoline-3-carboxylic acid	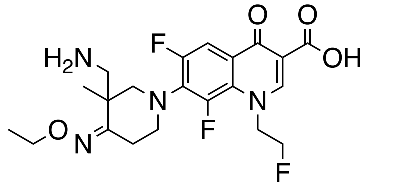	Idem
Inact32	(*Z*)-7-(3-(aminomethyl)-4-(ethoxyimino)-3-methylpiperidin-1-yl)-1-cyclopropyl-6-fluoro-8-methoxy-4-oxo-1,4-dihydroquinoline-3-carboxylic acid	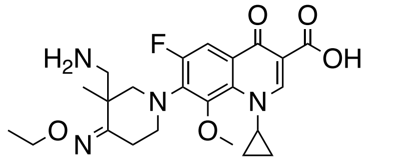	Idem
Inact33	7-(4-cyclopentylpiperazin-1-yl)-1-ethyl-6-fluoro-4-oxo-1,4-dihydroquinoline-3-carboxylic acid	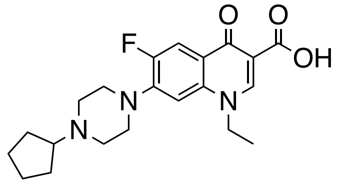	Abuo-Rahma Gel-D, Sarhan HA, Gad GF. Design, synthesis, antibacterial activity and physicochemical parameters of novel N-4-piperazinyl derivatives of norfloxacin. Bioorg Med Chem. 2009; 17(11):3879-3886.
Inact34	7-(4-(5,6-diphenyl-1,2,4-triazin-3-yl)piperazin-1-yl)-1-ethyl-6-fluoro-4-oxo-1,4-dihydroquinoline-3-carboxylic acid	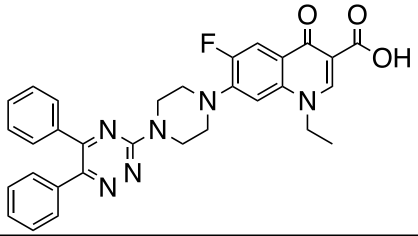	Idem
Inact35	7-(4-((dimorpholinoamino)methyl)piperazin-1-yl)-1-ethyl-6-fluoro-4-oxo-1,4-dihydroquinoline-3-carboxylic acid	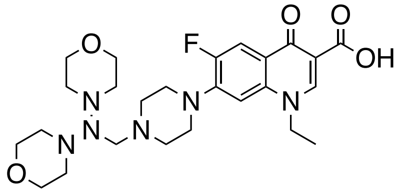	Idem

Inact36	7-(4-((bis(1,3-dioxoisoindolin-2-yl)amino)methyl)piperazin-1-yl)-1-ethyl-6-fluoro-4-oxo-1,4-dihydroquinoline-3-carboxylic acid	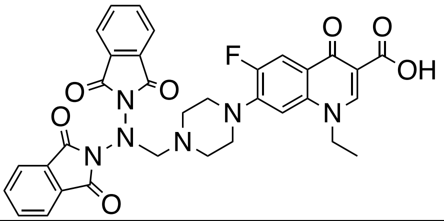	Idem
PGE-5215205	1-methyl-4-oxo-1,4-dihydroquinoline-3-carboxylic acid	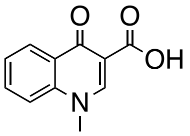	Macinga DR, Renick PJ, Makin KM, et al. Unique biological properties and molecular mechanism of 5,6-bridged quinolones. Antimicrob Agents Chemother. 2003; 47(8):2526-2537.
PGE-6116532	7,8-dimethyl-10-oxo-7,10-dihydro-1*H*-[1,3]dioxino[5,4-*f*]quinoline-9-carboxylic acid	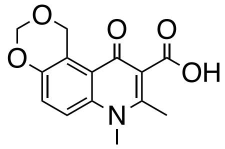	Idem
PGE-9604297	1-ethyl-4-oxo-1,4-dihydroquinoline-3-carboxylic acid	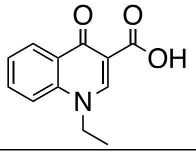	Idem
