# Supplementary material for: Tree-Based QSAR Model for Drug Repurposing in the Discovery of New Antibacterial Compounds against Escherichia coli
Source: Pharmaceuticals (Basel). 2020 Nov 28;13(12):431. doi: 10.3390/ph13120431 (PMC7760995; doi:10.3390/ph13120431)
Supplement: Supplementary file 1 [file pharmaceuticals-13-00431-s001.zip › Supplementary Material B Suay-Garcia/Supp Info 3 Table 3S-6S.pdf]

Table 3S. Results obtained in the LDA and compound classification according to the DF. Active training group.

Compound	PHIA	SH_{tCH}	$S_{=N-}$	$S_{>N-}$	$^{4}C_{C}$	FD	Prob.	Clas.
Grepafloxacin	4.269	15.1468	0	3.8448	0	25.2710	1.000	+
Norfloxacin	4.3595	14.4949	0	3.6034	0	14.5309	1.000	+
Clinafloxacin	4.0096	14.7581	0	3.4961	0	25.0192	1.000	+
Enoxacin	4.312	14.4324	0	3.3811	0	15.4349	1.000	+
Levofloxacin	4.245	14.9837	0	5.8165	0	12.9508	1.000	+
Lomefloxacin	4.7868	15.1927	0	2.9493	0	22.1550	1.000	+
Moxifloxacin	4.6366	15.3202	0	3.803	0	22.2894	1.000	+
Pradofloxacin	4.367	15.2491	0	3.608	0	26.5334	1.000	+
Ulifloxacin	3.9662	14.6175	0	3.9006	0	21.3927	1.000	+
Finafloxacin	4.3445	15.2083	0	3.4517	0	27.0421	1.000	+
Garenoxacin	5.0117	15.3699	0	1.4562	0	28.9818	1.000	+
Sparfloxacin	4.6719	15.6453	0	3.0307	0	30.9007	1.000	+
Act3	5.1308	14.9072	3.8853	3.1561	0	24.7741	0.935	+
Act4	5.1308	14.9396	3.9569	3.1511	0	25.5773	1.000	+
Act5	5.6007	15.0058	4.075	3.17	0	19.3139	1.000	+
Act7	4.6775	14.8281	3.8632	3.0849	0	31.1697	1.000	+
Act8	5.1308	14.8943	3.9813	3.1038	0	25.1767	1.000	+
Act32	5.6061	15.8019	4.0354	3.0286	1.1544	2.6638	1.000	+
Gemifloxacin	4.8944	14.8745	3.9688	3.3757	0	27.2736	1.000	+
Trovafloxacin	4.1573	14.835	0	2.731	0	27.7648	1.000	+
Act2	5.3424	15.1956	3.8779	2.9715	0	26.8901	1.000	+
Act14	6.0344	15.1822	4.0616	2.9802	0	16.0129	1.000	+
Act15	6.3432	15.381	4.2133	2.9739	0	14.7588	1.000	+
Act16	5.0862	14.8447	3.8649	2.9339	0	25.5575	1.000	+
Act18	5.5543	14.9433	4.0472	2.9478	0	20.1002	1.000	+
Act20	4.6348	14.7656	3.8354	2.8627	0	31.8956	1.000	+
Act21	5.0862	14.8318	3.9535	2.8816	0	25.9337	1.000	+
Act22	5.4263	15.0306	4.1052	2.8753	0	24.1686	1.000	+

Table 4S. Results obtained in the LDA and compound classification according to the DF. Inactive training group.

Compound	PHIA	*SHtCH*	*S(=N-)*	*S(>N-)*	C4C	FD	Prob.	Clas.
Inact33	5.422	14.8998	0	6.2612	0	-9.8488	1.000	-
Inact30	6.772	15.6554	4.1352	2.8651	1.1544	-17.4963	1.000	-
Inact32	6.5855	15.565	4.2361	3.6054	1.1544	-19.3399	1.000	-
Inact34	7.2905	15.3334	0	5.6188	0	-30.0179	1.000	-
Inact3	7.8915	15.6887	0	5.0073	0	-30.9581	1.000	-
Inact18	6.5842	15.207	0	5.7227	0	-21.0793	1.000	-
Inact23	7.1667	15.0976	0	5.5345	0	-31.3682	1.000	-
Inact31	7.2549	15.6693	4.1046	2.4621	1.1544	-23.1873	1.000	-

Table 5S. Results obtained in the LDA and compound classification according to the DF. Active test group.

Compound	PHIA	*SHtCH*	*S(=N-)*	*S(>N-)*	C4C	FD	Prob.	Clas.
Ciprofloxacin	3.8037	14.6077	0	3.7812	0	24.4927	1.000	+
Besifloxacin	4.8997	15.0234	0	3.5742	0	14.3929	1.000	+
Gatifloxacin	4.6898	15.1023	0	3.6897	0	18.4924	1.000	+
Ofloxacin	4.245	14.9837	0	5.8165	0	12.9508	0.998	+
Fleroxacin	5.237	15.1834	0	4.5829	0	6.3164	1.000	+
Act6	5.9211	15.2046	4.2268	3.1637	0	17.8711	1.000	+
Act9	5.4689	15.0931	4.133	3.0975	0	23.4452	1.000	+
Act1	5.8123	15.2747	3.8967	3.0427	0	20.2123	1.000	+
Act13	5.5562	15.116	3.9435	2.9613	0	22.4116	1.000	+
Act17	5.0862	14.8771	3.9292	2.9289	0	26.3346	1.000	+
Act19	5.8769	15.1421	4.199	2.9415	0	18.6215	1.000	+

Table 6S. Results obtained in the LDA and compound classification according to the DF. Inactive test group.

Compound	PHIA	*SHtCH*	*S(=N-)*	*S(>N-)*	C4C	FD	Prob.	Clas.
Inact6	6.448	15.1333	0	9.9826	0	-41.8344	1.000	-
