# Supplementary material for: Tree-Based QSAR Model for Drug Repurposing in the Discovery of New Antibacterial Compounds against Escherichia coli
Source: Pharmaceuticals (Basel). 2020 Nov 28;13(12):431. doi: 10.3390/ph13120431 (PMC7760995; doi:10.3390/ph13120431)
Supplement: Supplementary file 1 [file pharmaceuticals-13-00431-s001.zip › Supplementary Material B Suay-Garcia/Supp Info 4.pdf]

Table 2. Theoretically active compounds against *E. coil* selected by the model.

DRUG	THERAPEUTIC USE	ACTIVITY AGAINST *E. coli*	REF.
Acyclovir	Antiviral	Not described	1
Azacytidine	Antineoplastic	Not described	1
Azathioprine	Anti-rheumatic	Not described	1
Bicuculine	GABA antagonist	Not described	1
Cyclouridine	Not described	Not described	-
Cinoxacin	Antibiotic	Active	1
Citarabine	Antineoplastic	Not described	1
Clobetasol propionate	Glucocorticoid	Not described	1
Clonazepam	Anti-convulsive	Not described	1
2'Deoxycytidina	Protein	Not described	1
D-biopterin	Growth factor	Not described	1
D-threo-Neopterin	Growth factor	Not described	1
Decitabine	Antineoplastic	Not described	1
Deflazacort	Glucocorticoid	Not described	1
Didanosine	Antiviral	Not described	1
Diflorasone	Glucocorticoid	Not described	1
Difluprednate	Anti-inflammatory	Not described	1
Doxofylline	Antitussive	Not described	1
DNQX	Glutamate receptor	Not described	2
Dyprophylline	Bronchodilator	Not described	1
Ellagic acid	Antioxidant	Not described	1
Emtricitabine	Antiviral	Not described	1
Enoxacin	Antibiotic	Active	1
Entecavir	Antiviral	Not described	1
2-Fluoroadenosina	Not described	Not described	-
5-Fluorouridina	Not described	Not described	-
Floxuridine	Antineoplastic	Not described	1
Flucloronide	Glucocorticoid	Not described	1
Fluconazole	Antifungal	Not described	1
Fludarabine	Antineoplastic	Not described	1
Fludroxycortide	Glucocorticoid	Not described	1
Flumazenil	Benzodiazepine antagonist	Not described	1
Flumetasone	Glucocorticoid	Not described	1
Flunisolide	Glucocorticoid	Not described	1
Fluocinolone acetonide	Glucocorticoid	Not described	1
Fluocinonide	Glucocorticoid	Not described	1
Fluticasone propionate	Glucocorticoid	Not described	1

Furazolidone	Anti-infective	Not described	1
Ganciclovir	Antiviral	Not described	1
Ginkolide C	Neuroprotector	Not described	3
Guanosine	Nucleoside	Not described	1
Halcinonide	Anti-inflammatory	Not described	1
Idelalisib	Antineoplastic	Not described	1
Isatoribine	Antiviral	Not described	1
Kifunensine	Mannosidase inhibitor	Not described	4
Lamivudine	Antiviral	Not described	1
Levosimendan	Cardiotonic	Not described	1
Lidorestat	Diabetic neuropathy	Not described	1
Loflazepate ethyl	Anxiolytic	Not described	5
Lomefloxacin	Antibiotic	Active	1
Myricetina	Antioxidant	Not described	1
Nafamostat	Anti-inflammatory	Not described	1
Nitrofurantoin	Antibiotic	Active	1
Ofloxacin	Antibiotic	Active	1
Olaparib	Antineoplastic	Not described	1
Oteracil	Antineoplastic	Not described	1
Pefloxacin	Antibiotic	Active	1
Penciclovir	Antiviral	Not described	1
Pidotimod	Immunomodulator	Not described	1
Pyrroloquinoline quinone	Coenzyme	Not described	6
Pomalidomide	Immunomodulator	Not described	1
Quercetin	Antioxidant	Not described	1
Ribavirin	Antiviral	Not described	1
Riboflavin	Vitamin	Not described	1
Riociguat	Pulmonary arterial hypertension	Not described	1
Sapropterin	Phenylketonuria	Not described	7
Sarafloxacin	Antibiotic	Active	1
SB220025	Antineoplastic	Not described	8
Taxifolin	Anti-inflammatory	Not described	1
Tazobactam	Antibiotic	Active	1
Triamcinolone	Glucocorticoid	Not described	1
Troxacitabine	Antineoplastic	Not described	1
Ulobetasol	Glucocorticoid	Not described	1
Uric acid	Not described	Not described	-
Zardaverine	Bronchodilator	Not described	1
Zebularine	Antineoplastic	Not described	1
Zenarestat	Diabetic neuropathy	Not described	9

Zidovudine	Antiviral	Not described	1
ZM-241,385	Alzheimer	Not described	10
62T278S1MX	Glutamate receptor	Not described	11
8258W48TBZ	Antineoplastic	Not described	12
9-a-L-Arabinofuranosylguanine	Not described	Not described	-
1-a-Arabinofuranosylcytosine	Not described	Not described	-
(2E)-N-(3-Nitrophenyl)-4-[3-(1H-1,2,4-triazol-1-ylmethyl)phenyl]-2(5H)-pyrimidinimine	Not described	Not described	-
3-[5-(1H-Imidazol-1-yl)-7-methyl-1H-benzimidazol-2-yl]-4-[(2-pyridinylmethyl)amino]-2(1H)-pyridinone	Diabetes	Not described	13
3-[(3-Fluoro-3'-methoxy-4-biphenylyl)carbamoyl]-2-thiophenecarboxylic acid	Not described	Not described	-
2-(1,3-Benzodioxol-5-yl)-5-[(3-fluoro-4-methoxybenzyl)sulfanyl]-1,3,4-oxadiazole	Not described	Not described	-
7-nitroindazole-2-carboximidamide	Neuroprotector	Not described	14
(7R)-6-Formyl-7-(1-methyl-1H-1,2,3-triazol-4-yl)-4,7-dihydro-1,4-thiazepine-3-carboxylic acid	Not described	Not described	-
6-(3,4-Dihydroxybenzyl)-3-éthyl-1-(2,4,6-trichlorophényl)-1,5-dihydro-4H-pyrazolo[3,4-d]pyrimidin-4-one	Not described	Not described	-
3'-oxo-adenosine	Not described	Not described	-
(6*S*)-5,6,7,8-tetrahydrobiopterin	Neuroprotector	Not described	15
2-Amino-7-methyl-6-oxo-9-pentofuranosyl-6,7-dihydro-1H-purin-9-ium	Not described	Not described	-
Quinonoid 7,8-tetrahydrobiopterin	Not described	Not described	-
8-hydroxy-2'-deoxyguanosine	Not described	Not described	-
5-(Hydroxymethyl)-5,6,7,8-tetrahydrotetrazolo[1,5-a]pyridine-6,7,8-triol	Not described	Not described	-
(2Z,5Z)-5-[(5-Ethyl-2-furyl)methylene]-2-{[(4-fluorophenyl)(1H-tetrazol-5-yl)methyl]imino}-1,3-thiazolidin-4-one	Not described	Not described	-
(20s)-19,20,21,22-tetrahydro-19-oxo-5h-18,20-ethano-12,14-etheno-6,10-metheno-18h-benz[d]imidazo[4,3-k][1,6,9,12]oxatriaza-cyclooctadecosine-9-carbonitrile	Not described	Not described	-
4-[(2,6-Difluorobenzoyl)amino]-N-(4-fluorophenyl)-1H-pyrazole-3-carboxamide	Not described	Not described	-

N-{3-[(4-Anilino-8-cyanopyrazolo[1,5-a][1,3,5]triazin-2-yl)amino]phenyl}acetamide	Not described	Not described	-
(3Z)-1-[(6-Fluoro-4H-1,3-benzodioxin-8-yl)methyl]-3-(hydroxyimino)-4-phenyl-1,3-dihydro-2H-indol-2-one	Not described	Not described	-
N-Cyclopropyl-3-{[1-(2,4-difluorophenyl)-7-methyl-6-oxo-6,7-dihydro-1H-pyrazolo[3,4-b]pyridin-4-yl]amino}-4-methylbenzamide	Not described	Not described	-
3-[6-Bromo-2-fluoro-3-(1H-pyrazolo[3,4-c]pyridazin-3-ylmethyl)phenoxy]-5-chlorobenzonitrile	Not described	Not described	-
(5S,6Z)-4,5-Difluoro-6-[(2-fluoro-4-iodophenyl)imino]-N-(2-hydroxyethoxy)-1,3-cyclohexadiene-1-carboxamide	Not described	Not described	-
N-{2-[(2R)-4-Amino-5,8-difluoro-1,2-dihydro-2-quinazolinyl]ethyl}-3-furamide	Not described	Not described	-
4-[(1S)-1-(3-Fluoro-4-methoxyphenyl)-2-(2-methoxy-5-nitrophenyl)ethyl]-1H-imidazol-2-amine	Not described	Not described	-
2,4-Dinitrophenyl 2-deoxy-2-fluoro-β-D-mannopyranoside	Not described	Not described	-
(5E,7S)-7-[4-Fluoro-2-(3-pyridinyl)phenyl]-5-(hydroxyimino)-4-methyl-5,6,7,8-tetrahydro-2-quinazolinamine	Not described	Not described	-
(3E)-5-Fluoro-1-[(6-fluoro-4H-1,3-benzodioxin-8-yl)methyl]-3-(hydroxyimino)-1,3-dihydro-2H-indol-2-one	Not described	Not described	-
1-(5-Bromo-2-pyridinyl)-3-[(1S,2S)-2-(6-fluoro-2-hydroxy-3-propionylphenyl)cyclopropyl]urea	Not described	Not described	-
(5R)-5-{[(2-Amino-3H-purin-6-yl)oxy]methyl}-2-pyrrolidinone	Not described	Not described	-
2,6-Diamino-8-[(1H-imidazol-2-ylsulfanyl)methyl]-4(1H)-quinazolinone	Not described	Not described	-
(7R)-7-(6,7-Dihydro-5H-cyclopenta[d]imidazo[2,1-b][1,3]thiazol-2-yl)-2,7-dihydro-1,4-thiazepine-3,6-dicarboxylic acid	Not described	Not described	-
2-Anilino-9-(4-hydroxybutyl)-1,9-dihydro-6H-purin-6-one	Not described	Not described	-

3-(5-Amino-7-oxo-3,7-dihydro-2H-[1,2,3]triazolo[4,5-d]pyrimidin-2-yl)benzoic acid	Not described	Not described	-
9-Butyl-8-(2,5-dimethoxybenzyl)-2-fluoro-9H-purin-6-amine	Not described	Not described	-
(2R,4S)-6-Fluoro-2',5'-dioxo-2,3-dihydrospiro[chromene-4,4'-imidazolidine]-2-carboxamide	Not described	Not described	-
1-(2-Deoxy-α-L-erythro-pentofuranosyl)cytosine	Not described	Not described	-
(1R,3S)-7,8-Dihydroxy-1-methoxy-3-methyl-10-oxo-4,10-dihydro-1H,3H-pyrano[4,3-b]chromene-9-carboxylic acid	Not described	Not described	-
(2-Amino-3-benzoylimidazo[1,2-a]pyridin-6-yl)(2,6-difluorophenyl)methanone	Not described	Not described	-
8-(2,5-Dimethoxybenzyl)-2-fluoro-9-(4-pentyn-1-yl)-9H-purin-6-amine	Not described	Not described	-
1-{(Z)-[(5-Nitro-2-furyl)methylene]amino}-2,4-imidazolidinedione	Not described	Not described	-
(6S)-2,6-Anhydro-3-deoxy-3-fluoro-6-[(1R,2R)-1,2,3-trihydroxypropyl]-L-galactonic acid	Not described	Not described	-
7(S),8(R)-Dihydrobiopterin	Not described	Not described	-
3-(2H-Tetrazol-5-ylmethyl)-5,6,7,8-tetrahydro[1]benzothieno[2,3-d]pyrimidin-4(3H)-one	Not described	Not described	-
N-ethyl-5'-carboxamido adenosine	Not described	Not described	-
2-((9H-Purin-6-ylthio)methyl)-5-chloro-3-(2-methoxyphenyl)quinazolin-4(3H)-one	Not described	Not described	-
7-[(1R,5S)-6-Amino-3-azabicyclo[3.1.0]hex-3-yl]-1-(2,4-difluorophenyl)-6-fluoro-4-oxo-1,4-dihydro-1,8-naphthyridine-3-carboxylic acid	Not described	Not described	-
2,3,5,6-Tetrafluoro-4-methoxybenzamida	Not described	Not described	-
4-{[4-(4-Fluoro-3-methylphenyl)-1,3-thiazol-2-yl]amino}-2-hydroxybenzoic acid	Not described	Not described	-
(2S)-N-[(3Z)-5-Cyclopropyl-3H-pyrazol-3-ylidene]-2-[4-(2-oxo-1-imidazolidinyl)phenyl]propanamide	Not described	Not described	-
2-Amino-N-(4-methyl-1,3-thiazol-2-yl)-5-[(4-methyl-4H-1,2,4-triazol-3-yl)sulfanyl]benzamide	Not described	Not described	-

{3-[(4,5,7-Trifluoro-1,3-benzothiazol-2-yl)methyl]-1H-pyrrolo[2,3-b]pyridin-1-yl}acetic acid	Not described	Not described	-
2-(4-Fluorophenyl)-N-{[3-fluoro-4-(1H-pyrrolo[2,3-b]pyridin-4-yloxy)phenyl]carbamoyl}acetamide	Not described	Not described	-

References

1. DrugBank. Available online: https://www.drugbank.ca
2. Paul, K.N.; Fukuhara, C.; Karom, M.; Tosini, G.; Albers, H.E. AMPA/kainite receptor antagonist DNQX blocks the acute increase of Per2 mRNA levels in most but not all areas of the SCN. *Brain Res Mol Brain Res* **2005**, *139*, 129-136. doi: 10.1016/j.molbrainres.2005.05.017
3. Liou, C.J.; Lai, X.Y.; Chen, Y.L.; Wang, C.L.; Wei, C.H.; Huang, W.C. Ginkgolide C Supresses Adipogenesis in 3T3-L1 Adipocytes via the aMPK Signaling Pathway. *Evid Based Complement Alternat Med* **2015**, epub: 2015:298635. doi: 10.1155/2017/4293429
4. Vallee, F.; Karaveg, K.; Herscovics, A.; Moremen, K.W.; Howell, P.L. Structural basis for catalysis and inhibitoion of N-glycan processing class I alpha 1,2-mannosidases. *J Biol Chem* **2000**, *275*, 41287-41298. doi: 10.1074/jbc.M006927200
5. Sakai, Y.; Namima, M. Inhibitory effect on 3H-diazepam binding and potentiating action on GABA of ethyl loflazepate, a new minor tranquilizer. *Jpn J Pharmacol* **1985**, *37*, 373-379.
6. Sawyer, J.S.; Beight, D.W.; Britt, K.S. et al. Synthesis and activity of new aryl- and heteroaryl-substituted 5,6-dihydro-4H-pyrrolo[1,2-b]pyrazole inhibitors of the transforming growth factor-beta type 1 receptor kinase domain. *Bioorg Med Chem Lett* **2004**, *14*, 3581-3584. doi: 10.1016/j.bmcl.2004.04.007
7. Camp, K.M.; Parisi, M.A.; Acosta, P.B.; et al. Phenylketonuria Scientific Review Conference: State of the science and future research needs. *Mol Gen Metabol* **2014,** *112*, 87-11. doi: 10.1016/j.ymgme.2014.02.013
8. Wang, Z.; Canagarajah, B.J.; Boehm, J.C.; et al. Structural basis of inhibitor selectivity in MAP kinases. *Structure* **1998**, *6*, 1117-1128.
9. Kinoshita, T.; Miyake, H.; Fuji, T.; Takakura, S.; Goto, T. The structure of human recombinant aldose reductase complexed with the potent inhibitor zenarestat. *Acta Crystallogr* **2002**, *58*, 61-626. doi: 10.1107/s0907444902002378
10. Jaakola, V.P.; Griffith, M.T.; Hanson, M.A.; et al. The 2.6 Angstrom Crystal Structure of a Human A2A Adenosine Receptor Bound to an Antagonist. *Science* **2008**, *31*, 1211-1217. doi: 10.1126/science.1164772
11. Marinho da Silva, S.; Carrettiero, D.C.; Chadi, D.R. Glutamate requires NMDA receptors to modulate alpha2 adrenoceptor in medulla oblongata cultured cells of newborn rats. *Neurosci Lett* **2014**, *564*, 83-88. doi: 10.1016/j.neulet.2014.02.003
12. Yan, X.; Hollis, T.; Svinth, M.; Day, P.; et al. Structure-based identification of a ricin inhibitor. *J Mol Biol* **1997**, *266*, 1043-1049. doi: 10.1006/jmbi.1996.0865
13. Kontopidis, G.; Mcinnes, C.; Pandalaneni, S.R.; et al. Differential Binding of Inhibitors to Active and Inactive Cdk2 Provides Insights for Drug Design. *Chem Biol* **2006**, *13*, 201. doi: 10.1016/j.chembiol.2005.11.011
14. Raman, C.S.; Li, H.; Martasek, P.; Southan, G.; Masters, B.S.; Poulos, T.L. Crystal structure of nitric oxide synthase bound to nitro indazole reveals a novel inactivation mechanism. *Biochemistry* **2001**, *40*, 13448-13455. doi: 10.1021/bi010957u
15. Raman, C.S.; Li, H.; Martasek, P.; et al. Crystal Structures of Bovine Endothelial Nitric Oxide Synthase Heme Domain Complexed with the Pterin Analogues. RCSB PDB.
