# Supplementary figures and images for: Tree-Based QSAR Model for Drug Repurposing in the Discovery of New Antibacterial Compounds against Escherichia coli
Source: Pharmaceuticals (Basel). 2020 Nov 28;13(12):431. doi: 10.3390/ph13120431 (PMC7760995; doi:10.3390/ph13120431)

## Probability distribution diagrams of the analyzed discrete indexes.

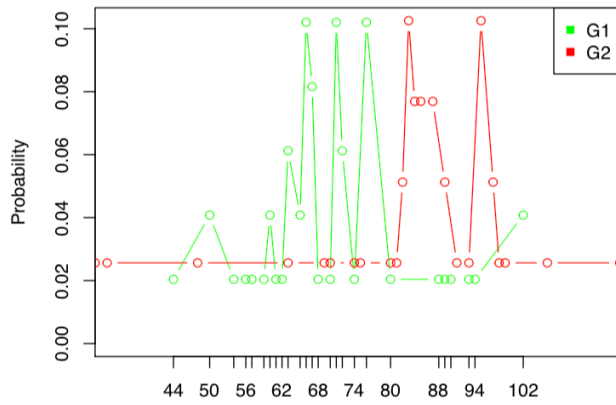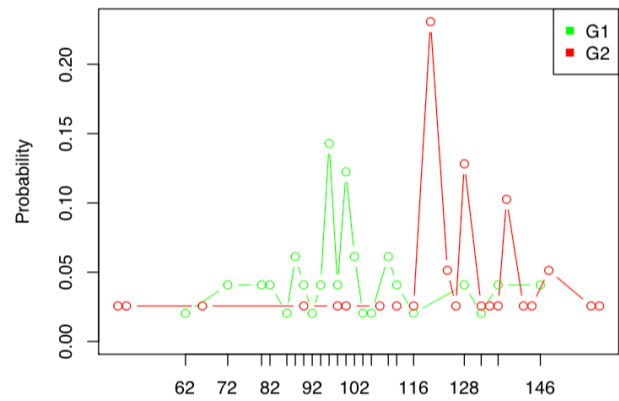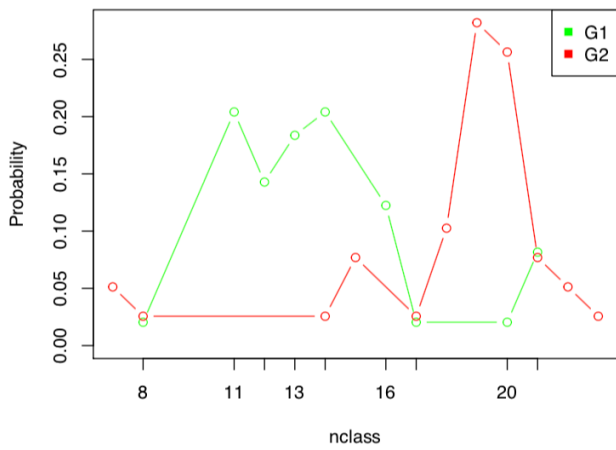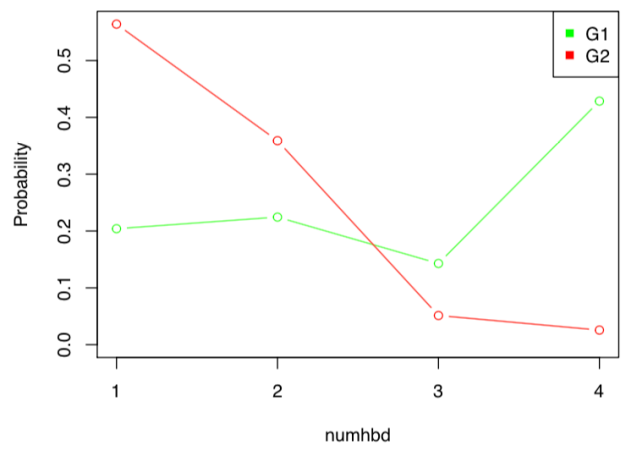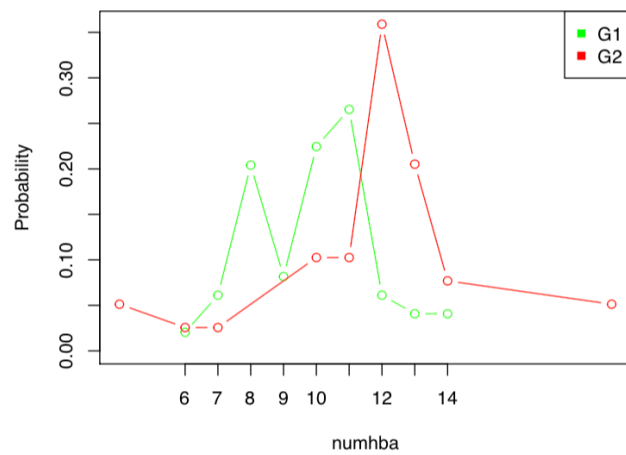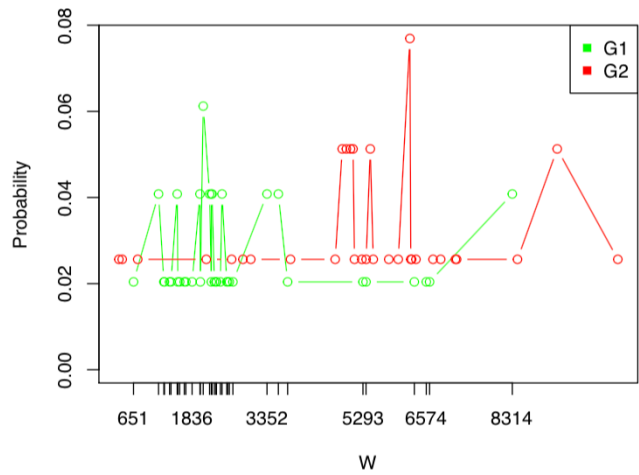

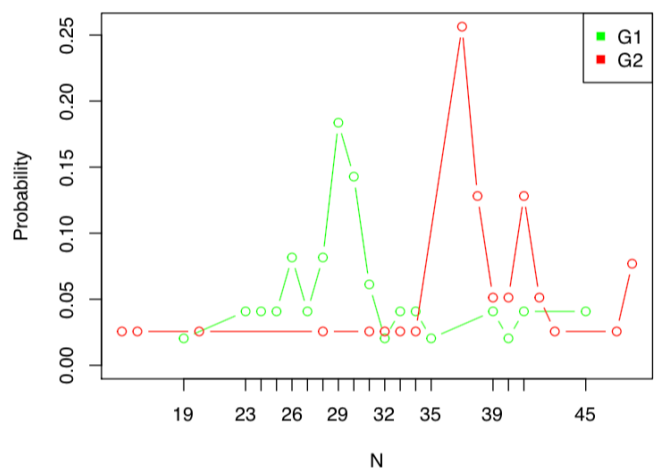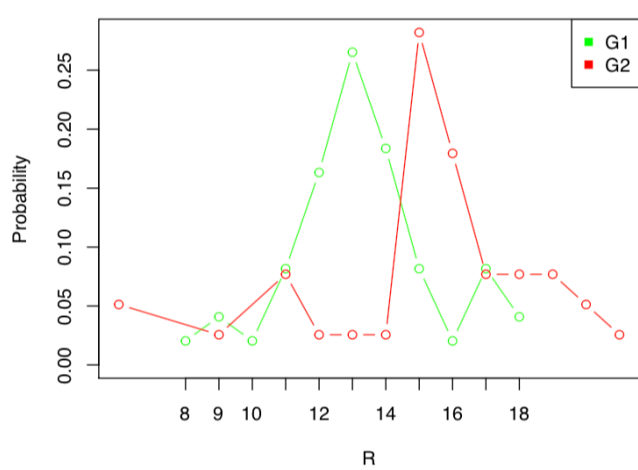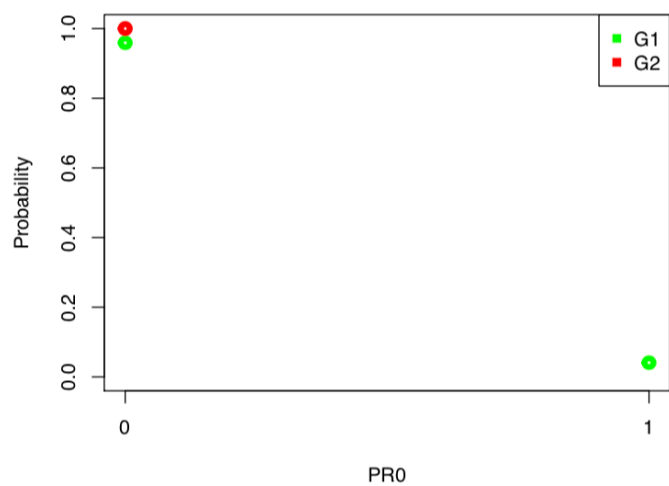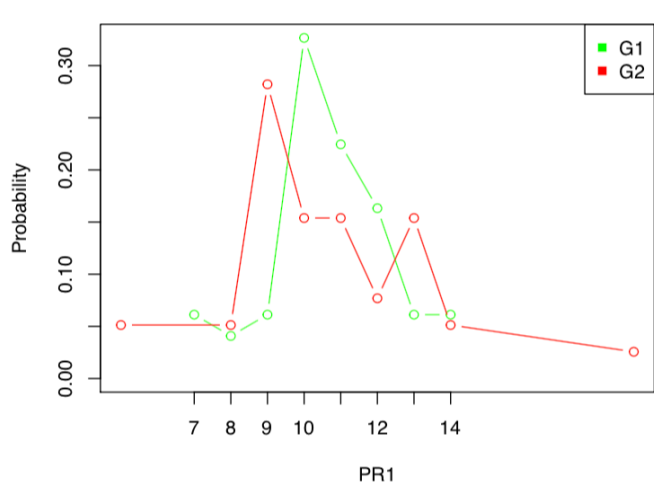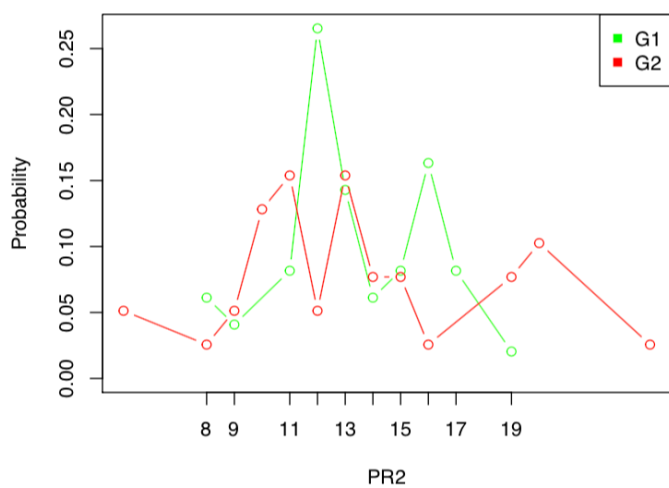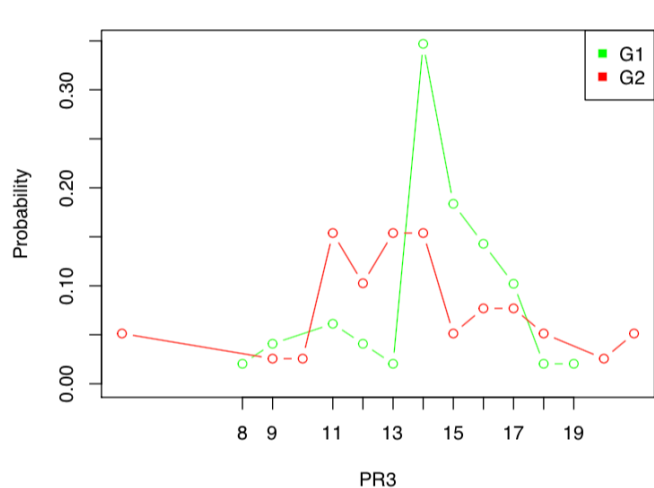

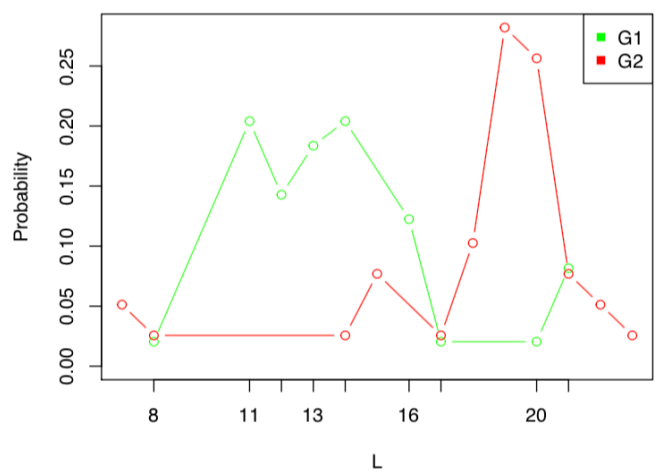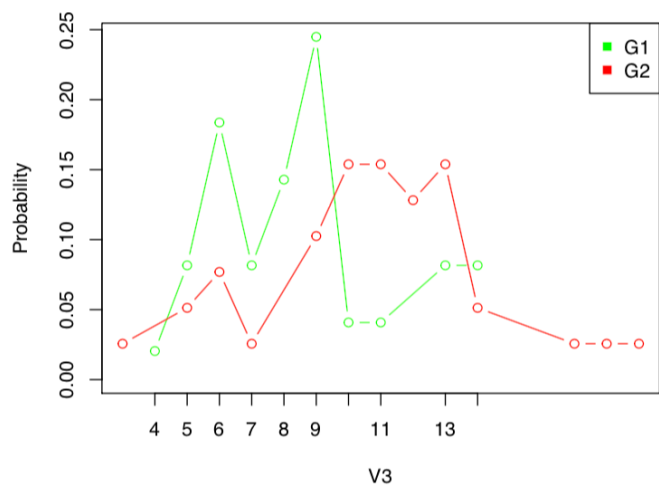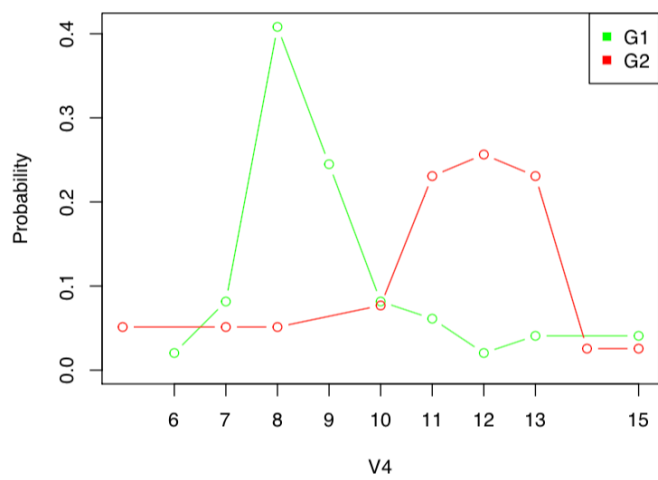

Supplement: Supplementary file 1 [file pharmaceuticals-13-00431-s001.zip › Supplementary Material B Suay-Garcia/Supp Info 2.pdf]
